# PGK1 Lactylation-Driven Self-Reinforcing Loop Orchestrates Glycolytic Reprogramming in FSP1^+^ Macrophages in Liver Fibrosis

**DOI:** 10.34133/research.1177

**Published:** 2026-03-03

**Authors:** Min Tang, Mengxue Sun, Hui Zhang, Jinwei Chen, Yuanben Wang, Yan Jiang, Linhua Qin, Hao Wang, Fengshang Zhu, Changqing Yang

**Affiliations:** ^1^ Department of Gastroenterology and Hepatology, Digestive Disease Institute, Tongji Hospital, Tongji University School of Medicine, Shanghai, China.; ^2^ School of Life Sciences and Technology, Tongji University, Shanghai, China.; ^3^ Department of Gastroenterology, Shanghai Tenth People’s Hospital, Tongji University School of Medicine, Shanghai, China.; ^4^ Department of Infectious Diseases, Shanghai General Hospital, Shanghai Jiao Tong University School of Medicine, Shanghai, China.; ^5^ Department of Gastroenterology, Luodian Hospital, Shanghai, China.; ^6^ Department of Critical Care Medicine, Shanghai Changzheng Hospital (Second Affiliated Hospital of Naval Medical University), Shanghai, China.

## Abstract

Liver fibrosis shows limited treatment efficacy, driven by metabolic reprogramming and epigenetics, while the role of lactate-mediated lactylation in hepatic microenvironment remains unclear. Here, through integrative analysis of public databases and human cirrhotic liver tissues, we identified a pathogenic FSP1^+^ (fibroblast specific protein 1) macrophage subset as a key therapeutic target. We uncovered a novel FSP1–glycolysis–lactylation axis that drives fibrotic progression through metabolic–immune crosstalk. Expanded FSP1^+^ macrophage infiltration was observed in human cirrhotic liver tissues and myeloid-specific Fsp1 knockout markedly attenuates inflammation and fibrosis. Mechanistic investigations reveal that FSP1 physically interacts with pyruvate kinase M2 (PKM2) in macrophages, inhibiting its ubiquitin–proteasome degradation to stabilize the enzyme. This FSP1–PKM2 interaction enhances glycolytic flux and lactate production, which in turn promotes KAT2B-dependent lactylation of phosphoglycerate kinase 1 (PGK1) at lysine 353 (K353). The posttranslational modification creates a positive feedback loop by concurrently activating PGK1 and pyruvate dehydrogenase kinase 1, which blocks mitochondrial pyruvate metabolism, thereby amplifying glycolysis and PGK1 lactylation. Notably, we developed a cell-penetrating peptide targeting PGK1-K353 lactylation that effectively attenuates the progression of liver fibrosis. Our findings establish lactate-mediated lactylation of PGK1 as a critical node in fibrotic metabolism and reveal a previously unrecognized FSP1–glycolysis axis that sustains the pro-fibrotic microenvironment. Targeting PGK1-K353 lactylation represents a promising therapeutic strategy for chronic liver diseases.

## Introduction

Liver fibrosis, particularly in its terminal stage, has long been considered incurable, with liver transplantation as the remaining sole definitive curative option [1–3]. As a dynamic pathological cascade, liver fibrosis is primarily driven by the persistent hyperactivation of hepatic inflammation [3]. This response transforms the self-limiting tissue repair process into a vicious cycle, thereby accelerating fibrotic progression [4–6].

Immune cells, especially recruited macrophages, are recognized as key drivers of hepatic inflammation, a central determinant governing the progression or resolution of liver fibrosis [2,5,6]. Activated macrophages are typically categorized into 2 phenotypes: classically activated proinflammatory (M1) and alternatively activated anti-inflammatory (M2) macrophages [7–9]. During early liver injury, bone marrow-derived monocytes (BMDMs) extensively infiltrate the liver, differentiating into inflammatory macrophages (predominantly M1), releasing proinflammatory cytokines that trigger hepatic inflammatory responses and activate hepatic stellate cells (HSCs) [10–12]. FSP1, a pivotal member of the S100 calcium-binding protein family, is considered a marker for a subgroup of macrophages [13]. Previous evidence demonstrates that FSP1 functions as a pro-fibrogenic paracrine factor, activating HSCs through specific cell surface receptors [13]. Although functional interactions between FSP1^+^ macrophages and HSCs have been experimentally validated [13], critical knowledge gaps persist regarding the precise molecular mechanisms and signaling networks that govern this pathological interaction.

Glycolysis plays a critical role in the classical activation of M1 macrophages, generating diverse metabolic intermediates supporting macrophage activation [14], while inevitably leading to the accumulation of metabolic end products like lactate [15,16]. Traditionally viewed as a terminal waste product of anaerobic glucose metabolism, lactate has recently been recognized to play multifaceted roles as a signaling molecule, energy substrate, and immunomodulatory factor [17]. Lactate-driven lactylation modifies histones/proteins, directly influencing mRNA splicing, glycolysis, and RNA transport. This posttranslational modification (PTM) links to inflammation, neurodevelopment, and tumorigenesis [18,19]. In liver fibrosis pathogenesis, lactylation may act as a bridge between metabolic reprogramming and gene dysregulation within fibrotic signaling networks [20]. However, the lactylation landscape in liver fibrosis remains poorly characterized, with its precise roles in fibrogenic initiation and progression largely unknown. Characterizing these mechanisms is essential for developing targeted therapies against fibrosis.

This study interrogates metabolic dysfunction, inflammatory signatures of FSP1^+^ hepatic macrophage subsets, and their impact on HSC activation during liver fibrogenesis. In human and murine fibrotic liver tissues, we observed a marked expansion of the FSP1^+^ macrophage population, coupled with increased global protein lactylation. Compared to wild-type (WT) mice, myeloid-specific Fsp1 knockout mice (*LysM^cre^Fsp1^f/f^*) exhibited attenuated pathological hallmarks in liver fibrosis. Mechanistically, multi-omics analysis revealed that FSP1 interacts with pyruvate kinase M2 (PKM2), a key rate-limiting enzyme in the glycolytic pathway [21], to enhance glycolytic flux, resulting in lactate accumulation within macrophages. Phosphoglycerate kinase 1 (PGK1), the first adenosine triphosphate (ATP)-generating enzyme in the glycolytic cascades [22], was identified as a key downstream effector. Lactylation of PGK1 at lysine 353 (K353) induced its hyperactivation and mitochondrial translocation, thereby activating pyruvate dehydrogenase kinase 1 (PDHK1) and suppressing mitochondrial pyruvate metabolism to sustain glycolytic dominance. This establishes a self-reinforcing glycolysis/PGK1-K353 lactylation axis in FSP1^+^ macrophages that exacerbates metabolic dysregulation, lactylation overload, and the vicious cycle of pro-fibrotic macrophage polarization. Furthermore, we designed a cell-penetrating peptide (CPP) specifically targeting PGK1 lactylation at the K353 site. This peptide potently suppresses M1 macrophage polarization, reduces inflammatory cell infiltration, and ultimately ameliorates liver fibrosis progression. Collectively, this study uncovers a novel regulatory mechanism linking FSP1-mediated metabolic reprogramming to lactylation-driven fibrogenesis and highlights PGK1-K353 lactylation as a potential therapeutic target to interrupt the pathological cascades of liver fibrosis.

## Results

### Up-regulation of FSP1 in macrophages drives pro-fibrotic inflammation in liver fibrosis

**Fig. 1.**
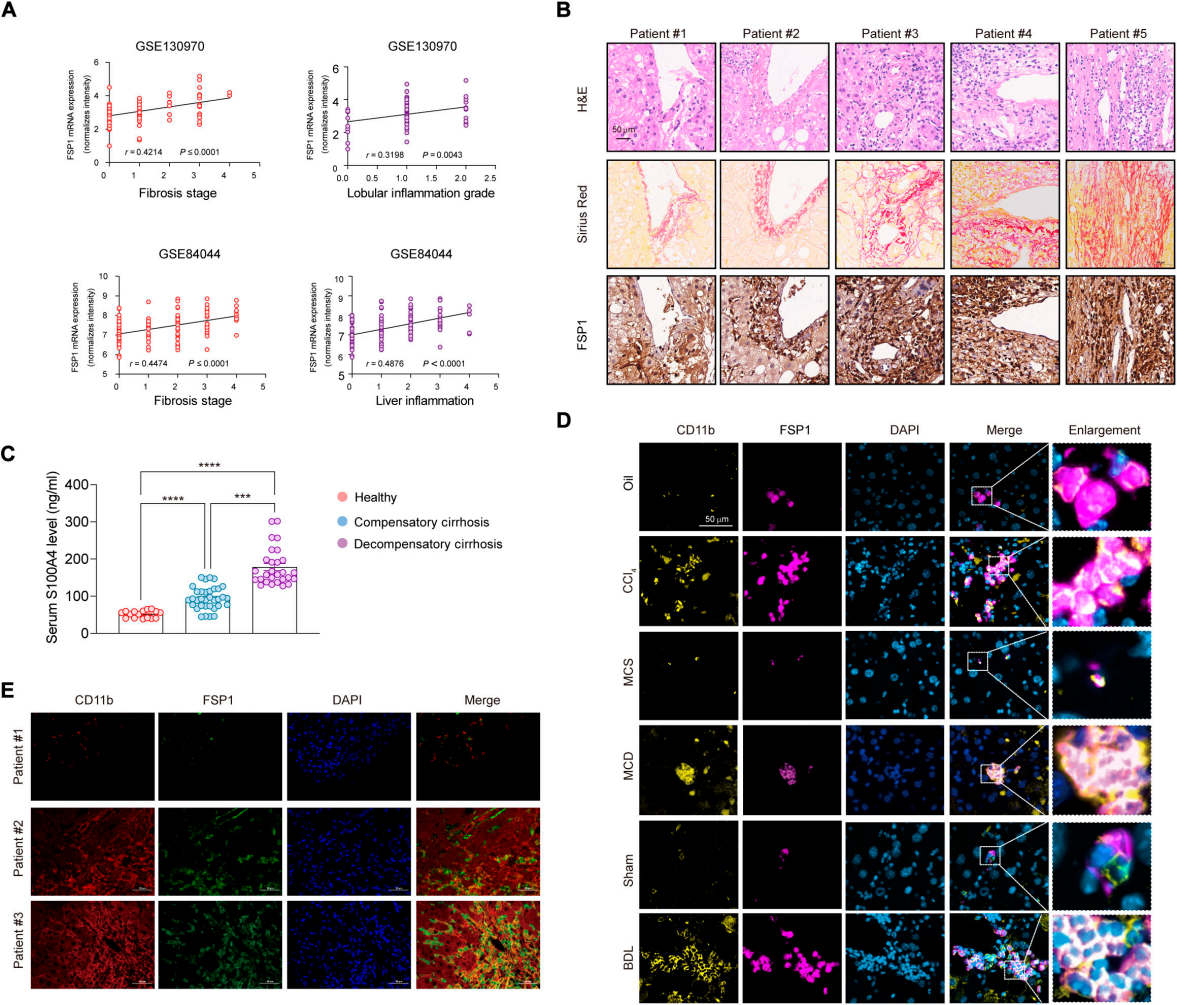
FSP1 expression is up-regulated in macrophages from the liver tissues of patients and mice models with liver fibrosis. (A) Correlation analysis of FSP1 expression levels with clinical parameters in GEO datasets related to liver fibrosis caused by diverse etiologies (GSE130970: MAFLD; GSE84044: HBV). (B) Representative images of H&E staining, Sirius Red staining and immunohistochemical (IHC) images of FSP1 staining in the cirrhotic liver tissue (Patient #2 to 5) and control (Patient #1). Scale bar, 50 μm. *n* = 20 for cirrhosis samples and *n* = 8 for the controls. (C) Serum FSP1 level in healthy donors (*n* = 16) and in compensated (*n* = 34) and decompensated (*n* = 28) cirrhotic patients detected by ELISA. (D) Dual-immunofluorescence staining for FSP1 and CD11b was performed on control and murine fibrotic liver samples induced by CCl_4_ injection (toxic injury), MCD diet (metabolic dysfunction), and BDL (cholestatic injury). Scale bar, 50 μm. *n* = 6 per group. (E) Representative dual-immunofluorescence staining of FSP1 and CD11b in liver samples from cirrhosis patient (Patient #3 and 5) and control (Patient #1). *n* = 20 for cirrhosis samples. Scale bar, 50 μm. Statistical significance was determined by one-way ANOVA, ***P < 0.001, ****P < 0.0001.

To identify key genes in fibrotic pathogenesis, we analyzed 3 independent Gene Expression Omnibus (GEO) datasets (GSE84044, GSE130970, and GSE183754) containing liver tissues from healthy controls and patients with fibrosis of various etiologies (Fig. S1A). Cross-dataset analysis identified 17 consistently up-regulated genes (Fig. S1B and C). Among them, FSP1 showed stage-dependent elevation during fibrosis progression and correlated positively with inflammatory signatures (Fig. 1A). Supporting its therapeutic potential, selective ablation of proliferating FSP1^+^ cells restored liver tissue architecture more effectively than targeting αSMA^+^ cells (Fig. S1D, GSE55747). Our results showed that FSP1 levels were elevated in fibrotic areas of cirrhotic livers (Fig. 1B), and serum FSP1 was elevated in patients (Fig. 1C). Previous studies have reported the expression of FSP1 in monocytes/macrophages [13]. Consistently, single-cell RNA sequencing (scRNA-seq) analysis showed that FSP1 is predominantly expressed in monocytes/macrophages (Fig. S1E). Its expression was markedly up-regulated in macrophages cells upon fibrotic progression (Fig. S1F). Co-staining and primary cell isolation further confirmed that FSP1 predominantly localized to macrophages in both murine and human fibrotic livers, with minimal expression in HSCs, hepatocytes, or endothelial cells (Fig. S1G to I). Consistent with human data, 3 mouse models of liver fibrosis induced by carbon tetrachloride (CCl_4_), a methionine-choline-deficient (MCD) diet, or bile duct ligation (BDL) all showed significant increases in myeloid FSP1 (Fig. 1D and E). Collectively, these results indicate that myeloid FSP1 expression significantly increases during liver fibrosis and correlates with its severity.

**Fig. 2.**
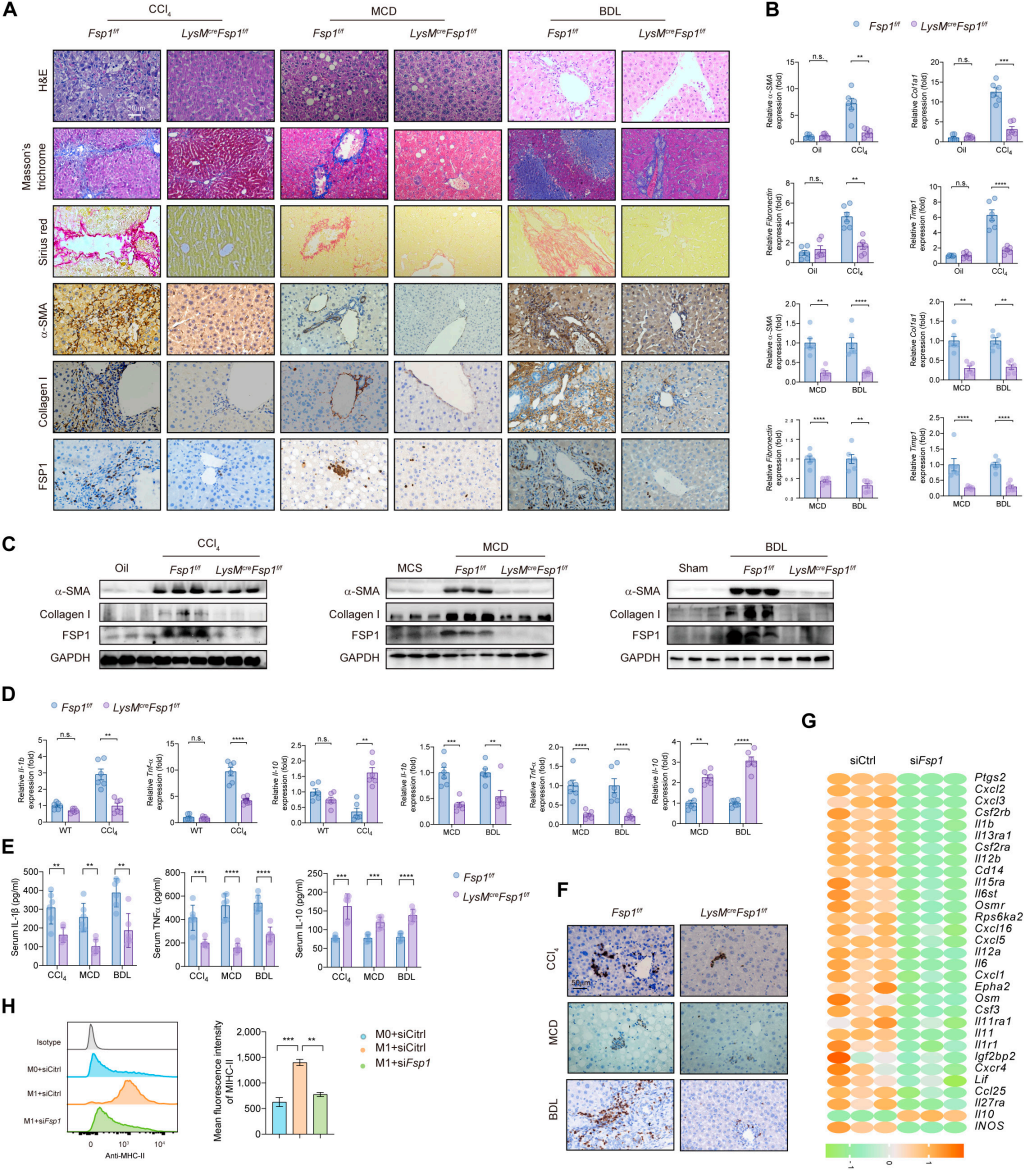
Myeloid-specific *Fsp1* knockout ameliorates liver fibrosis. (A) *Fsp1^f/f^* and *LysM^cre^Fsp1^f/f^* mice were induced to liver fibrosis by CCl_4_ injection (toxic injury), MCD diet (metabolic dysfunction), and BDL (cholestatic injury). The degree of liver fibrosis was evaluated by H&E, Masson’s trichrome staining, Sirius Red, and IHC staining images of alpha-smooth muscle actin (α-SMA) and collagen I staining and FSP1 staining. Scale bar, 100 μm in the CCl_4_ group and 50 μm in the MCD and BDL group. (*n* = 6 per group). (B) The mRNA expression levels of *α-SMA*, *Col1α1*, *Fibronectin*, and tissue inhibitor of metalloproteinase 1 (*Timp-1*) were quantified by quantitative reverse transcription polymerase chain reaction (qRT-PCR) in liver tissues isolated from the mice in (A). (C) Protein expression of α-SMA, collagen I, and FSP1 was analyzed by Western blot in liver samples from indicated groups. (D) The mRNA expression levels of proinflammatory [interleukin (IL)-1β and tumor necrosis factor (TNF)-α] and anti-inflammatory (IL-10) cytokine were quantified in liver tissues isolated from the indicated groups. *n* = 6 per group. (E) Serum IL-1β, TNF-α, and IL-10 were measured by ELISA. *n* = 6 per group. (F) IHC staining of Ly6G were detected in the indicated groups, Scale bar, 50 μm. (G) Heatmap showing the relative expression of inflammatory factors in M1-polarized BMDM transfected with siFSP1. *n* = 3 per group. (H) Mean fluorescence intensity (MFI) of MHC-II in BMDMs, as detected by flow cytometry. *n* = 3. Data were presented as mean ± SEM. Statistical significance was determined by unpaired Student *t* test (B, D, and E) or one-way ANOVA (H). **P < 0.01, ***P < 0.001, ****P < 0.0001. ns, no significant difference.

To assess the function of macrophage FSP1 in liver fibrosis, we generated myeloid-specific FSP1 knockout (*LysM^cre^Fsp1^f/f^*) mice. The *LysM^cre^Fsp1^f/f^* mice exhibited attenuated collagen deposition across all fibrosis models (Fig. 2A to C), with no observable effects in healthy mice (Fig. S2A). We investigated the inflammatory role of FSP1 in liver fibrosis by profiling cytokine expression and immune cell infiltration. *LysM^cre^Fsp1^f/f^* fibrotic mice showed reduced proinflammatory cytokines (tumor necrosis factor-α [TNF-α] and interleukin-1β [IL-1β]) and elevated anti-inflammatory IL-10 (Fig. 2D and E), accompanied by diminished Ly6G^+^ neutrophil infiltration (Fig. 2F), indicating that FSP1^+^ macrophages drive proinflammatory responses in liver fibrosis. In vitro, FSP1-deficient BMDMs displayed lowered mRNA levels of proinflammatory cytokines and increased anti-inflammatory cytokines upon lipopolysaccharide (LPS)/interferon-γ (IFN-γ) stimulation (Fig. 2G and Fig. S2B), together with impaired M1 polarization (Fig. 2H), consistent with reduced inducible nitric oxide synthase (iNOS) expression in *LysM^cre^Fsp1^f/f^* fibrotic livers (Fig. S2C). In coculture with HSCs, FSP1-deficient BMDMs attenuated HSC activation, suppressing *α-SMA* and *Col1a1* expression (Fig. S2D and E).

### FSP1 promotes glycolysis in macrophages by directly interacting with PKM2 and enhancing its stability

**Fig. 3.**
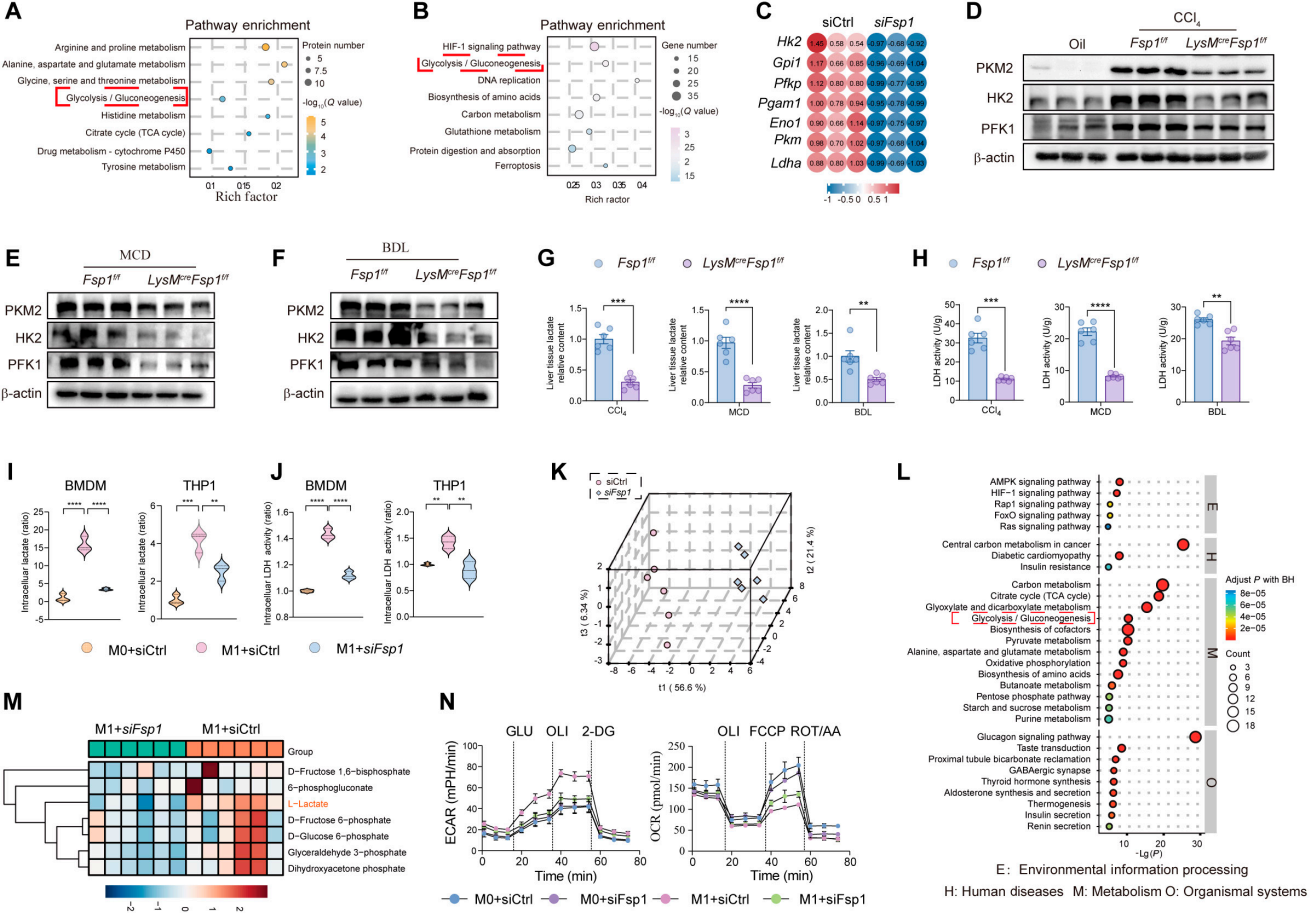
FSP1 induces macrophage glycolytic reprogramming during hepatic fibrogenesis. (A) A comparative KEGG analysis of DEPs between *Fsp1f/f* and *LysMcreFsp1f/f* mice highlighted metabolic reprogramming, such as in glycolysis/gluconeogenesis, following CCl_4_ injury. *n* = 3 per group. (B) Total RNA from BMDMs was subjected to RNA-sequencing analysis. KEGG pathway analysis of differentially expressed genes (DEGs) revealed metabolic alterations, including glycolysis/gluconeogenesis, in M1-polarized BMDMs transfected with *siFsp1* or negative control siRNA (siCtrl). *n* = 3 per group. (C) Heatmaps displaying DEGs associated with glycolysis, as defined in (B). (D to F) Protein expression of PKM2, HK2, and PFK1 was analyzed by Western blot in control liver samples and fibrotic liver samples from *Fsp1f/f* and *LysM^cre^Fsp1^f/f^* mice induced by CCl_4_ injection (D), MCD diet (E), or BDL (F). (G) Measurement of lactate production in fibrotic tissue from the mice in (D) to (F). *n* = 6 per group. (H) Measurement of LDH activity in fibrotic tissue from the mice in (D) to (F). *n* = 6 per group. (I) THP-1 monocytes were differentiated to M0 macrophages with phorbol 12-myristate 13-acetate (PMA). Intracellular lactate was measured in BMDMs and PMA-differentiated THP1 receiving siCtrl and *siFsp1*. *n* = 3 per group. (J) LDH activity was measured in BMDMs and PMA-differentiated THP1 receiving siCtrl and *siFsp1*. *n* = 3 per group. (K) PLS-DA of targeted metabolomics analyses on FSP1-editing in M1-polarizated BMDMs was performed to explore the impact of FSP1 on the metabolism of BMDMs. *n* = 6 per group. (L) Metabolic pathways were enriched in significantly altered metabolites identified by KEGG analysis. (M) Heatmap shows changes of glycolysis-related metabolites. The metabolite (L-lactate) with a statistically significant change in levels is marked in red. (N) The extracellular acidification rate (ECAR, left panel) and the oxygen consumption rate (OCR, right panel) in BMDM with the indicated treatments.

To elucidate the mechanisms of FSP1-mediated hepatic fibrogenesis, we performed proteomic analysis of CCl_4_-treated *Fsp1^f/f^* and *LysM^cre^Fsp1^f/f^* mice. Kyoto Encyclopedia of Genes and Genomes (KEGG) pathway analysis revealed significant enrichment of glycolysis-related pathways (Fig. 3A). RNA sequencing of LPS/IFN-γ-stimulated M1 macrophages after *Fsp1* knockdown confirmed pronounced glycolytic remodeling and reduced expression of key energy metabolism enzymes (Fig. 3B and C). Validation studies showed elevated expression of rate-limiting glycolytic enzymes (HK2, PKM2, and PFK1) in fibrotic livers compared to healthy controls, which was attenuated in *LysM^cre^Fsp1^f/f^* mice (Fig. 3D to F). Similarly, M1-polarized BMDMs and THP1 macrophages showed up-regulation of these enzymes, reversible upon FSP1 knockdown (Fig. S3A and B). Fibrotic livers exhibited increased lactate production and LDH activity, both suppressed by myeloid-specific FSP1 knockout (Fig. S3C and Fig. 3G and H). In BMDMs, M1 polarization-induced lactate accumulation and LDH activity were reversed by FSP1 knockdown (Fig. 3I and J), identifying FSP1 as a key regulator of glycolytic reprogramming in macrophage-driven fibrogenesis. Targeted metabolomics of FSP1-knockdown BMDMs showed distinct metabolic profiles via PLS-DA and PCA (Fig. 3K and Fig. S3D), with 7 metabolites up-regulated and 18 down-regulated (Fig. S3E). KEGG enrichment indicated glycolytic pathway disruption (Fig. 3L), supported by reduced levels of glycolytic intermediates (D-fructose 6-phosphate, glyceraldehyde 3-phosphate, L-lactate) (Fig. 3M). Functional assessment using Seahorse assays revealed that FSP1 knockdown reversed the M1-polarization-induced increase in extracellular acidification rate (ECAR; glycolysis) and decrease in oxygen consumption rate (OCR; oxidative phosphorylation) (Fig. 3N), confirming that FSP1 deficiency attenuates the M1 macrophage glycolytic switch.

**Fig. 4.**
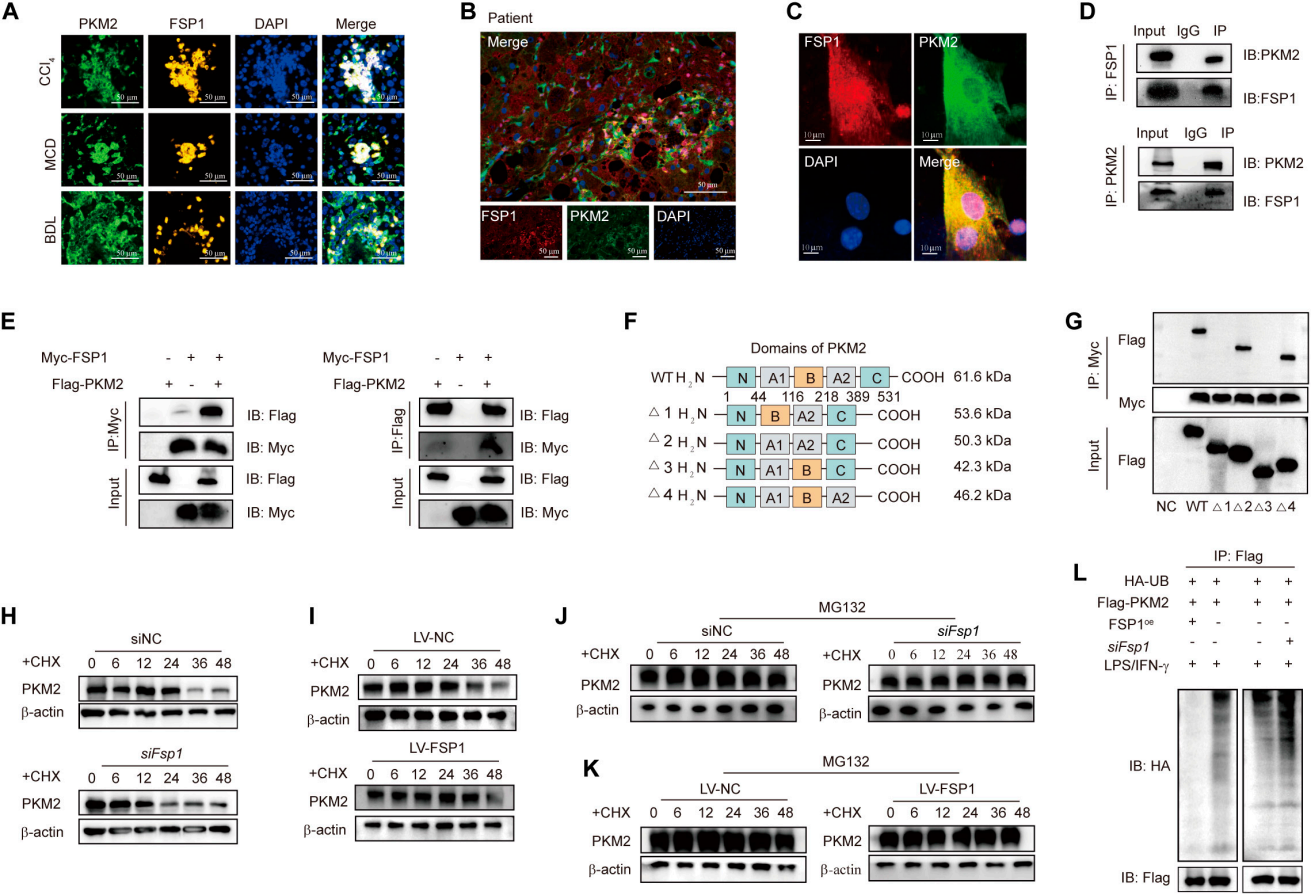
FSP1 binds to PKM2 and stabilizes it by inhibiting ubiquitin-mediated proteasomal degradation. (A) Dual-immunofluorescence staining of FSP1 and PKM2 in 3 murine models of liver fibrosis induced by CCl_4_ injection, MCD diet, and BDL. *n* = 6 per group. Scale bar, 50 μm. (B) Dual-immunofluorescence staining of FSP1 and PKM2 in cirrhosis patients. *n* = 20. Scale bar, 50 μm. (C) Representative immunofluorescence images show that FSP1 colocalizes with PKM2 in BMDMs, with signals detected in both the cytoplasm and nucleus. Scale bar, 10 μm. *n* = 3 technical replicates per group. (D) Representative immunoblots of FSP1 and PKM2 in a co-immunoprecipitation (Co-IP) assay of BMDMs. IgG indicates the control antibody group. *n* = 3 technical replicates per group. (E) Representative immunoblots showing the interaction between Flag-tagged PKM2 and Myc-tagged FSP1 in an IP assay of HEK293T cells. *n* = 3 technical replicates per group. (F) The predicted molecular weights of each protein are indicated. A schematic of the domain architecture and predicted molecular weights for Flag-tagged PKM2 truncation mutants (Δ1 to Δ4) and the full-length WT protein. (G) Representative immunoblots of the various constructs of Flag-PKM2 illustrated in in a Co-IP assay of HEK293T for Myc-FSP1 and Flag-PKM2 WT or mutant (top) and the overall expression levels of the various Flag-PKM2 constructs in these cells (bottom). *n* = 3 technical replicates per group. (H) M1-polarized BMDMs isolated from WT mice and transfected with siCtrl or *siFsp1* were treated with cycloheximide (CHX, 100 ng/ml) for the indicated time periods. *n* = 3 technical replicates per group. (I) BMDMs transfected with lentiviruses overexpressing FSP1 (LV-FSP1) or lentivirus-negative control (LV-NC) were treated with CHX (100 ng/ml) for the indicated time periods. *n* = 3 technical replicates per group. (J) BMDMs isolated from WT mice were transfected with siCtrl or *siFsp1* and polarized to M1 phenotype. The cells were treated with CHX (100 ng/ml) for the indicated time periods, and MG132 (50 μM) was added to the cell supernatant 6 h before sample collection. *n* = 3 technical replicates per group. (K) BMDMs transfected with LV-FSP1 or LV-NC were treated with CHX (100 ng/ml) for the indicated time periods. MG132 (50 μM) was added to the cell supernatant 6 h before sample collection. *n* = 3 technical replicates per group. (L) In vitro ubiquitination assay was performed in M1 BMDMs, *siFsp1*-transfected M1 BMDMs, and FSP1-overexpressing (OE) M1 BMDMs. Cells were cotransfected with Flag-PKM2 and HA-UB plasmids. Cell lysates were immunoprecipitated with an anti-Flag magnetic beads, followed by immunoblot analysis using anti-Flag antibodies. *n* = 3 technical replicates per group. All data were mean ± SEM. *P < 0.05, **P < 0.01, ***P < 0.001, ****P < 0.0001 by Student *t* test (G and H) or one-way ANOVA (I and J).

We observed elevated expression of mitochondrial complex III in FSP1-deficient macrophages (Fig. S3F). Since PKM2 inhibits complex III activity in macrophages [23] and bioinformatic analysis suggested an FSP1–PKM2 interaction (Fig. S3G), we hypothesized that FSP1 regulates macrophage energy metabolism via PKM2. Immunofluorescence in fibrotic mouse and human livers, along with confocal imaging in BMDMs, confirmed FSP1–PKM2 colocalization in cytoplasmic and nuclear compartments (Fig. 4A to C and Fig. S3H). Co-immunoprecipitation (Co-IP) in BMDMs and transfected human embryonic kidney 293T (HEK293T) cells validated their physical interaction (Fig. 4D and E). To map the FSP1–PKM2 interaction interface, we generated PKM2 domain deletion mutants (Δ1: A1 domain-deleted; Δ2: B domain-deleted; Δ3: A2 domain-deleted; Δ4: C domain-deleted) (Fig. 4F). Co-IP revealed that Δ1 and Δ3 mutants lost binding capacity to FSP1 (Fig. 4G), identifying the A1 and A2 domains as critical interaction interfaces. FSP1 knockdown did not affect PKM2 mRNA (Fig. S3I) but shortened its protein half-life, while FSP1 overexpression prolonged PKM2 stability (Fig. 4H and I). Proteasome inhibition with MG-132 restored PKM2 levels in FSP1-deficient cells (Fig. 4J and K), and ubiquitination assays confirmed that FSP1 suppresses PKM2 ubiquitination and degradation (Fig. 4L). To elucidate the structural mechanism by which FSP1 binding shields PKM2 from ubiquitination, we performed comparative molecular dynamics simulations between the FSP1–PKM2 complex (System 1) and the TRIM21–PKM2 complex (System 2), with TRIM21 being a known E3 ligase for PKM2 [24]. Our analysis revealed that the FSP1–PKM2 complex exhibited superior global conformational stability (lower root-mean-square deviation [RMSD]), imposed specific rigidity on PKM2 regions 50 to 220 and 290 to 320 (reduced root-mean-square fluctuation [RMSF]), and maintained a more robust intermolecular hydrogen-bond network compared to the TRIM21–PKM2 complex (Fig. S3J to M). Crucially, Molecular Mechanics/Poisson–Boltzmann Surface Area (MM/PBSA) binding free energy calculations demonstrated a profoundly favorable Δ*G*_bind_ for the FSP1–PKM2 interaction, in stark contrast to the unfavorable binding energy for TRIM21–PKM2 (Fig. S3N to P). These structural and energetic data support a model wherein FSP1, by forming a highly stable complex, competitively occludes the binding interface for E3 ligases such as TRIM21 on PKM2, thereby explaining the observed suppression of PKM2 ubiquitination and degradation. These results establish FSP1 as a key posttranslational stabilizer of PKM2, modulating macrophage glycolysis during fibrogenesis.

To investigate glycolysis as a therapeutic target for liver fibrosis, we administered the glycolytic inhibitor 2-deoxyglucose (2-DG) in murine fibrosis model. 2-DG treatment significantly reduced hepatic proinflammatory cytokine expression while increasing anti-inflammatory factors (Fig. S4A to C), paralleled by decreased serum cytokine levels (Fig. S4D to F). Fibrosis-related gene expression (Fig. S4G to I), collagen deposition (Fig. S4J), and collagen protein levels (Fig. S4K) were markedly suppressed after 2-DG treatment. Additionally, 2-DG attenuated neutrophil infiltration, iNOS expression (Fig. S4J and K), and HSC activation (Fig. S4L). These findings provide compelling evidence for glycolytic reprogramming as a viable therapeutic strategy against liver fibrosis.

### FSP1-induced lactate accumulation drives PGK1 lactylation at K353 by KAT2B lactyltransferase

**Fig. 5.**
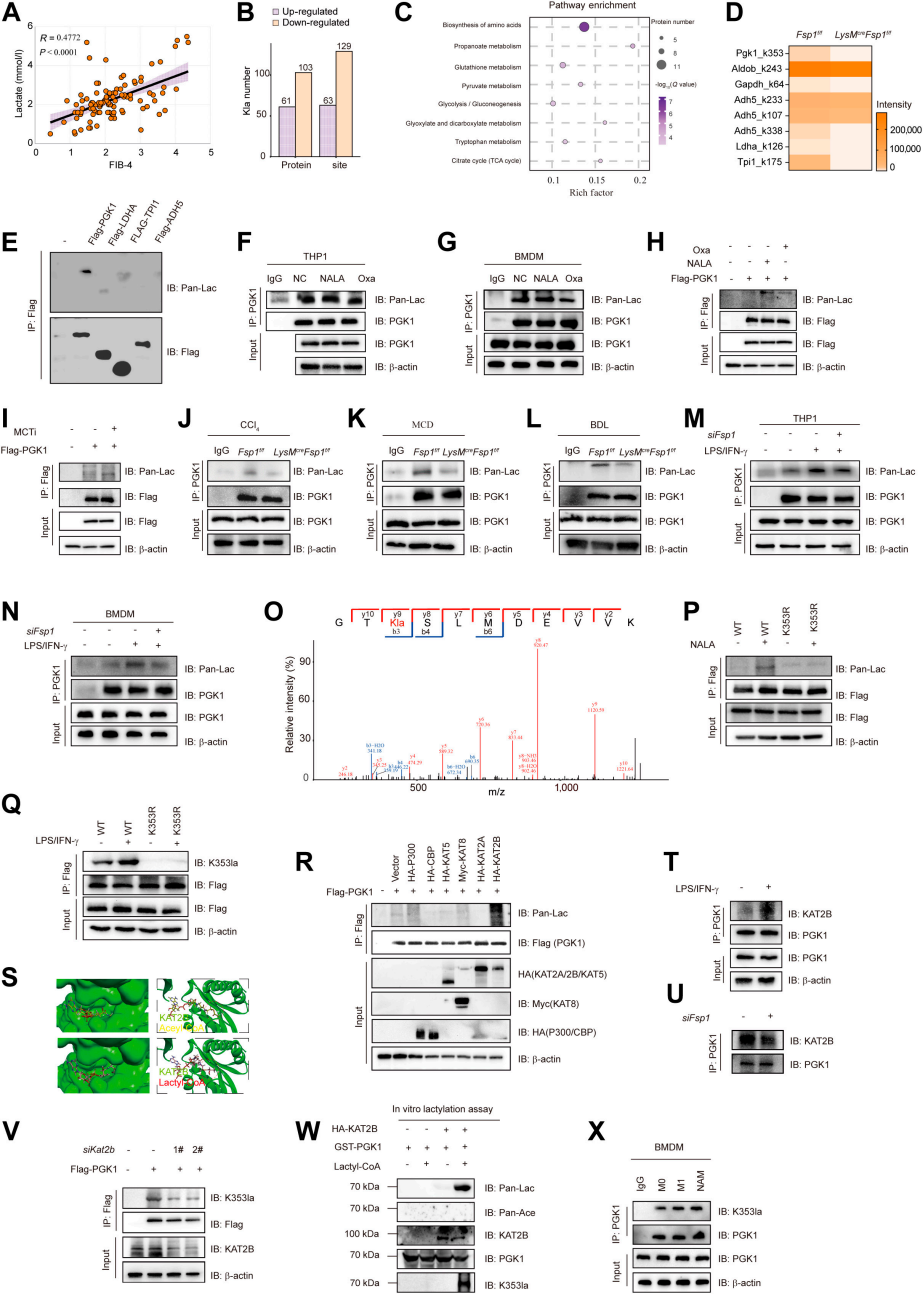
FSP1-induced lactate accumulation drives PGK1 lactylation at K353 by KAT2B lactyltransferase. (A) Relationship of FIB4 and serum lactate from cirrhosis patients (*n* = 102). (B) *Fsp1f/f* and *LysM^cre^Fsp1^f/f^* mice were subjected to CCl_4_-induced hepatic fibrosis modeling (*n* = 3 per group). Liver tissues were collected, and whole protein lysates were prepared. Immunoprecipitation (IP) was performed using pan-lactylated lysine antibodies, followed by mass spectrometry (MS) analysis to identify lactylated proteins. The graph depicts the number of proteins with significantly altered lactylation levels between fibrotic *Fsp1f/f* and *LysM^cre^Fsp1^f/f^* livers, determined by quantitative MS-based proteomics. (C) Pathway enrichment of DEPs using KEGG database defined as in (B). (D) Heatmap depicting lactylation levels of lysine residues in glycolysis-related proteins with statistically significant changes. (E) Detecting lactylation of the proteins identified in (D) by Western blot using IP sample as indicated. *n* = 3 technical replicates per group. (F and G) PMA-differentiated THP1 (F) cells and BMDMs (G) were treated for 24 h with NALA or LDHi (Oxamate [Oxa]) and induced to an M1 phenotype. Cell lysates were immunoprecipitated with an anti-PGK1 antibody, followed by immunoblot analysis using anti-Pan-Lac or anti-PGK1 antibodies. *n* = 3 technical replicates per group. (H) HEK293T cells transfected with Flag-tagged PGK1 plasmid were treated with NALA or Oxa. IP was performed to detect the lactylation modification levels of PGK1. *n* = 3 technical replicates per group. (I) HEK293T cells were pretreated with 20 mM MCT1/4i for 12 h prior to transfection with a Flag-tagged PGK1 plasmid. Detecting lactylation of PGK1 using IP samples as indicated. *n* = 3 technical replicates per group. (J to L) Detection of PGK1 lactylation in *Fsp1f/f* and *LysM^cre^Fsp1^f/f^* fibrotic liver tissues induced by CCl_4_ injection (J), MCD diet (K), and BDL (L) using the indicated IP samples. IgG serves as the control antibody group. *n* = 3 technical replicates per group. (M and N) The lactylation of PGK1 was measured in IP samples from PMA-differentiated THP1 (M) and BMDMs (N) that were transfected with *siFsp1* and polarized to the M1 phenotype. *n* = 3 technical replicates per group. (O) Illustration of PGK1 K353 lactylation identified by MS. (P) PGK1 lactylation assay was performed in HEK293T cells transfected with Flag-tagged WT-PGK1 or K353R-PGK1 plasmids, followed by treatment with 25 mM NALA for 24 h. *n* = 3 technical replicates per group. (Q) BMDMs were transduced with lentiviral vectors encoding WT PGK1 or the K353R mutant to establish stable overexpression, followed by induction of M1 macrophage polarization. Lactylation of PGK1 was subsequently assessed in IP lysates using Western blot analysis with the indicated antibodies. *n* = 3 technical replicates per group. (R) Screening the “writer(s)” of PGK1 lactylation by transfecting combined Flag-tagged PGK1 and acetyltransferase as indicated. (S) The cofactor binding pockets of KAT2B (PDB ID: 4NSQ) are shown in complex with acetyl-CoA (top panel) and lactyl-CoA (bottom panel). KAT2B is depicted as a cartoon model, and the transferable moieties in acetyl-CoA or lactyl-CoA are marked with red circles. (T and U) Co-IP showing interactions between KAT2B and PGK1 in BMDMs. *n* = 3 technical replicates per group. (V) Lactylation detection of PGK1 was performed via IP in HEK293T cells that had been transfected with Flag-tagged PGK1 plasmid and treated with siRNA targeting KAT2B (*siKat2b*), as evidenced by Western blot analysis. *n* = 3 technical replicates per group. (W) Detecting PGK1 lactylation using an in vitro lactylation assay on the indicated samples. *n* = 3 technical replicates per group. (X) Detecting lactylation of PGK1 in BMDMs treated with NAM using IP samples as indicated. *n* = 3 technical replicates per group.

The biological functions of cells can be influenced by various metabolites through metabolic modification of the proteome [25]. Lactylation, a recently discovered form of PTM, remains poorly understood in the context of hepatic fibrosis. We began by examining the relationship between nonhistone protein lactylation and intracellular lactate dynamics in macrophages. Supplementation with sodium lactate (NALA) increased global lactylation levels, while inhibition of glycolysis (2-DG) or lactate dehydrogenase (LDH; oxamate [Oxa]) reduced them (Fig. S5A to C), indicating that lactate accumulation directly promotes lactylation. Since FSP1 enhances glycolytic lactate production in fibrotic livers, we measured serum lactate in cirrhotic patients and found it elevated and positively correlated with fibrosis severity (*r* = 0.4772, *P* < 0.0001; Fig. 5A). We therefore hypothesized that FSP1 influences fibrogenesis by modulating protein lactylation. Western blot analysis showed increased global lactylation and LDHA expression in fibrotic mouse livers (Fig. S5D). LDHA expression and overall lactylation levels were reduced in the liver from *LysM^cre^Fsp1^f/f^* mice (Fig. S5D). In BMDMs from multiple fibrotic models, myeloid-specific FSP1 knockout similarly decreased LDHA and lactylation (Fig. S5E). LPS/IFN-γ stimulation enhanced LDHA expression and lactylation, and effects were reversed by FSP1 knockdown (Fig. S5F to H).

Proteomic analysis revealed extensive reprogramming of the protein lactylation landscape in fibrotic livers from *Fsp1^f/f^* and *LysM^cre^Fsp1^f/f^* mice (Fig. S6A and B). We identified 549 differentially lactylated proteins, showing heterogeneous subcellular distribution and involvement in diverse biological processes (Fig. S6C to E). Myeloid-specific FSP1 knockout significantly reduced lactylation of 103 proteins (Fig. 5B), many enriched in metabolic pathways such as glycolysis (Fig. 5C). Focus analysis of glycolysis-related proteins identified PGK1 as a key lactylation substrate (Fig. 5D and E). NALA treatment enhanced PGK1 lactylation, whereas lactate dehydrogenase inhibitor (LDHi) had the opposite effect (Fig. 5F to H). Inhibiting lactate export with MCT1/4i increased intracellular lactate and PGK1 lactylation (Fig. S6F and Fig. 5I). These findings suggest that FSP1 promotes hepatic fibrosis partly through PGK1 lactylation. To validate PGK1 as a key lactylation target in hepatic fibrosis, we confirmed its elevated lactylation in fibrotic livers (Fig. S7A), while it is reduced in the liver from *LysM^cre^Fsp1^f/f^* mice (Fig. 5J to L). M1 polarization-induced lactylation was reversed by FSP1 silencing (Fig. 5M and N). LC-MS identified K353 as the primary lactylation site, conserved across species (Fig. 5O and Fig. S7B). A K353R mutation abolished lactylation (Fig. 5P), and a monoclonal antibody (PGK1-K353la) confirmed the specificity of this modification (Fig. S7C). In BMDMs overexpressing WT or K353R PGK1, M1 polarization increased PGK1-K353la signal only in WT cells (Fig. 5Q). Furthermore, the PGK1 K353R mutant abrogated the ability of M1 macrophages to activate HSCs in coculture (Fig. S7D and E), indicating that FSP1-dependent lactylation of PGK1 at K353 promotes fibrogenesis.

To dissect the regulatory mechanisms of PGK1 lactylation, we investigated enzymatic and nonenzymatic pathways [26,27]. Inhibiting GLO1, a regulator of nonenzymatic lactylation, did not alter PGK1 lactylation (Fig. S7F). Through acetyltransferase screening, we identified PCAF (KAT2B) as a putative lactyltransferase (Fig. 5R). Molecular docking suggested that lactyl-CoA binds to the KAT2B cofactor pocket in a conformation analogous to acetyl-CoA (Protein Data Bank [PDB]: 4NSQ; Fig. 5S). Co-IP confirmed KAT2B–PGK1 interaction in HEK293T cells (Fig. S7G and H), which was enhanced by M1 polarization or lactate supplementation and attenuated by FSP1 knockdown (Fig. 5T and U and Fig. S7I). Genetic or pharmacological inhibition of KAT2B reduced PGK1 lactylation (Fig. 5V and Fig. S7J), corroborated by in vitro lactylation assays (Fig. S7K and Fig. 5W). Furthermore, the sirtuin inhibitor nicotinamide (NAM) increased PGK1 lactylation (Fig. 5X and Fig. S7L and M), suggesting that sirtuins may function as delactylases. To explore the potential “eraser” enzyme responsible for PGK1 delactylation, we conducted a candidate screening. This finding identifies SIRT7 as a strong candidate for the delactylase that may regulate PGK1-K353 lactylation dynamics (Fig. S7N). Together, these results define an FSP1-driven, KAT2B-mediated lactylation of PGK1 at K353 that promotes liver fibrosis.

### Glycolysis/PGK1 lactylation forms a positive feedback loop in FSP1^+^ macrophages in hepatic fibrosis

**Fig. 6.**
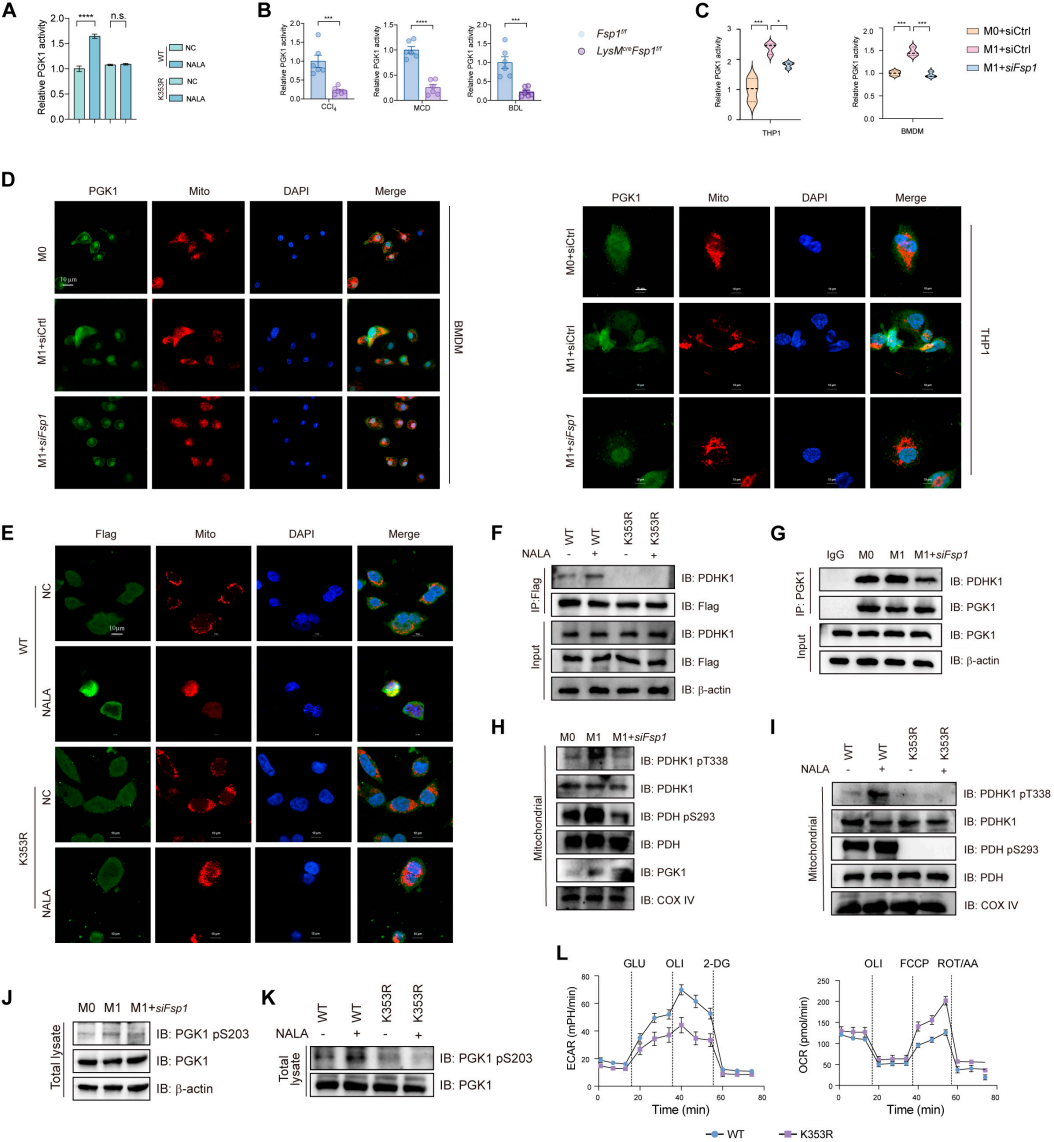
Glycolysis/PGK1 lactylation forms a positive feedback loop in FSP1^+^ macrophages of mice with hepatic fibrosis. (A) Enzymatic activity of PGK1 was assessed in HEK293T cells transfected with Flag-tagged PGK1 WT or K353R point mutant construct, followed by treatment with NALA. *n* = 3 per group. (B) The enzymatic activity of PGK1 was measured in liver tissues isolated from *Fsp1^f/f^* and *LysM^cre^Fsp1^f/f^* mice with liver fibrosis induced by CCl_4_ injection, MCD diet, or BDL. *n* = 6 per group. (C) The enzymatic activity of PGK1 was measured in PMA-differentiated THP-1 and BMDMs with the indicated treatment. *n* = 3 biological replicates. (D) BMDMs and THP1 transfected with *siFsp1* or siCtrl were polarized with M1-inducing stimuli, followed by triple staining with anti-PGK1 antibody (green), MitoTracker (red), and DAPI (blue). Scale bar, 50 μm. *n* = 3 biological replicates. (E) Representative immunofluorescence images of HEK293T cells were transfected with Flag-tagged PGK1 WT or K353R mutant construct, under untreated (NC) or stimulated with NALA (50 mM, 24 h) conditions. Cells were costained with anti-Flag antibody (green, PGK1 localization) and MitoTracker Red (mitochondrial marker). Nuclear counterstaining was performed with DAPI (blue). Scale bar, 50 μm. *n* = 3 biological replicates. (F) PDHK1 expression was analyzed by IP in HEK293T cells expressing Flag-PGK1 WT or its K353R point mutant construct. *n* = 3 biological replicates. (G) BMDMs transfected with *siFsp1* or siCtrl were polarized with M1-inducing cytokines. Whole-cell lysates were subjected to IP using anti-PGK1 antibody, followed by immunoblotting for PDHK1. *n* = 3 biological replicates. (H) Immunoblotting analysis of PDHK1 and PDH phosphorylation in mitochondria. Mitochondrial fractions were prepared from BMDMs and immunoblotted with indicated antibodies. *n* = 3 biological replicates. (I) PDHK1 and PDH phosphorylation was detected in mitochondria by immunoblotting analysis. Mitochondrial fractions were prepared from HEK293T cells receiving Flag-tagged PGK1 WT or K353R point mutant construct. *n* = 3 biological replicates. (J) PGK1 phosphorylation was detected in BMDMs with the indicated treatment. *n* = 3 biological replicates. (K) PGK1 phosphorylation was detected in HEK293T cells transfected with Flag-tagged PGK1 WT or K353R point mutant construct, and followed by NALA treatment. *n* = 3 biological replicates. (L) BMDMs transfected with lentiviruses overexpressing WT PGK1 or the K353R mutant were polarized to M1 phenotype. ERCR and OCR were measured to assess glycolysis and mitochondrial oxidative phosphorylation, respectively. *n* = 5 to 6 per group. Data were presented as mean ± SEM; Statistical significance was determined by unpaired Student *t* test (A and B) or one-way ANOVA (C). *P < 0.05, ***P < 0.001, ****P < 0.0001. ns, no significant difference.

PGK1 catalyzes the first ATP-generating step in glycolysis and is critical for coordinating energy production with biosynthesis and redox homeostasis [22,28]. To assess the functional consequences of K353 lactylation on PGK1, we conducted in vitro enzymatic assays. Enhancing cellular lactylation with NALA increased PGK1 activity, an effect abolished by the K353R mutation (Fig. 6A). FSP1 deficient in macrophages significantly reduced PGK1 glycolytic activity (Fig. 6B and C). PGK1 has been reported to mediate its mitochondrial translocation through S203 phosphorylation [29]. Upon translocation into mitochondria, PGK1 acts as a protein kinase to phosphorylate and activate PDHK1, which subsequently phosphorylates and inhibits the activity of pyruvate dehydrogenase (PDH). This process blocks mitochondrial pyruvate metabolism and promotes glycolysis [22]. We next examined whether PGK1 K353 lactylation regulates its mitochondrial translocation and metabolic function. Confocal microscopy showed that M1 polarization markedly enhanced PGK1-mitochondria colocalization in macrophages, which was abolished by FSP1 knockdown (Fig. 6D). In HEK293T cells expressing Flag-PGK1, NALA-induced lactylation promoted mitochondrial translocation of WT PGK1, but not the K353R mutant (Fig. 6E). Co-IP assays revealed that lactylation strengthened PGK1–PDHK1 interaction in both HEK293T cells with NALA and M1 macrophages, an effect abolished by the K353R mutation or FSP1 knockdown (Fig. 6F and G). Cellular fractionation confirmed that M1 polarization increased mitochondrial PGK1 translocation and subsequent phosphorylation of PDHK1 T338 and PDH S293, which was reversed by FSP1 down-regulation (Fig. 6H). Similarly, in HEK293T cells, NALA enhanced these phosphorylation events in a K353-dependent manner (Fig. 6I). Since mitochondrial translocation of PGK1 has been linked to S203 phosphorylation [22], we found that M1 polarization or NALA treatment induced S203 phosphorylation in WT PGK1, but not in the K353R mutant (Fig. 6J and K), suggesting that K353 lactylation facilitates S203 phosphorylation-dependent mitochondrial translocation. To gain structural insight into how K353 lactylation promotes S203 phosphorylation, we performed molecular dynamics simulations comparing unmodified (System 1) and K353-lactylated PGK1 (System 2). The lactylated protein exhibited increased global conformational flexibility and a marked enhancement in local flexibility around the S203-proximal region (residues 202 to 204) (Fig. S8A and B). Visualization of the simulation trajectories revealed that K353 lactylation induces an outward displacement of the S203 side chain by approximately 2.4 Å toward the solvent-accessible surface (Fig. S8C). These data suggest that lactylation-driven conformational changes increase the exposure and accessibility of S203, potentially facilitating its recognition and phosphorylation by upstream kinases. Functionally, the K353R mutant elevated OCR and reduced ECAR compared to WT (Fig. 6L), indicating a metabolic shift toward oxidative phosphorylation. These results reveal a feedforward loop in FSP1^+^ macrophages wherein FSP1-driven glycolysis promotes PGK1 K353 lactylation, which in turn enhances mitochondrial PDHK1/PDH signaling and reinforces glycolytic reprogramming during fibrogenesis.

**Fig. 7.**
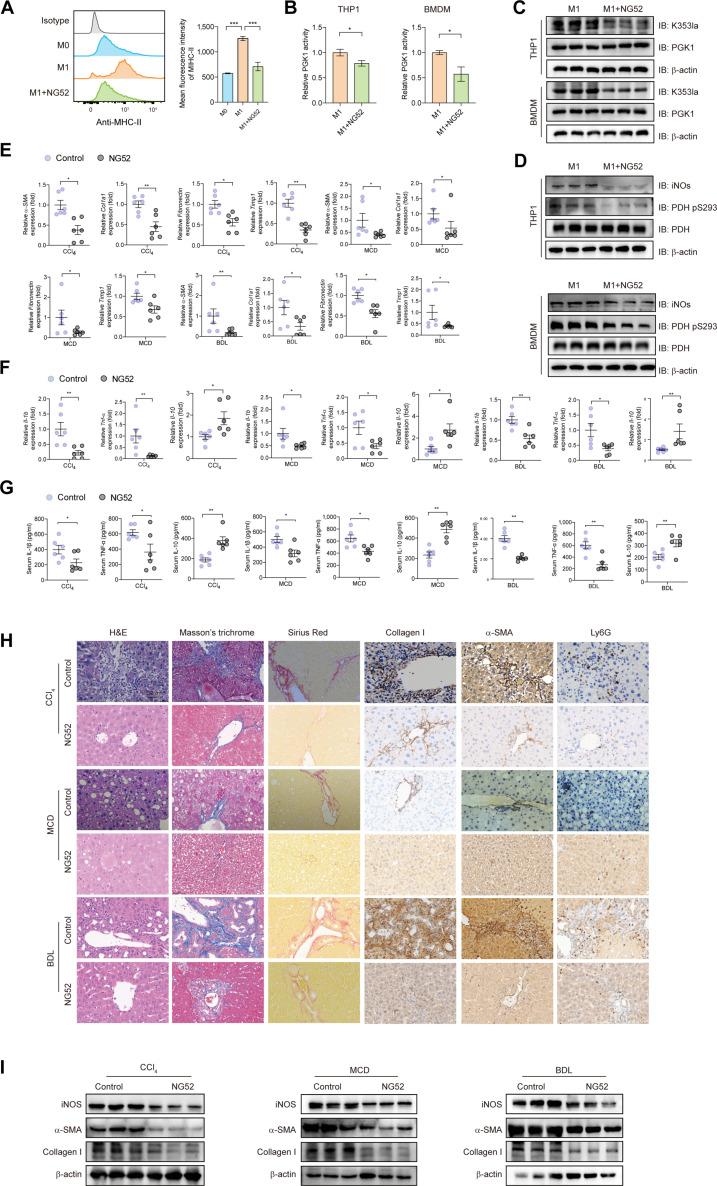
PGK1 inhibition prevents the progression of liver fibrosis. (A) The MFIs of MHC-II were detected in BMDMs subjected to NG52 (a PGK1 inhibitor, 25 μM, 24 h) treatments after M1 induction, as determined by flow cytometry. *n* = 3 biological replicates. (B) The enzymatic activity of PGK1 was assessed in the indicated groups. *n* = 3 biological replicates. (C) THP1-derived macrophages and BMDMs were polarized to M1 phenotype, followed by treatment with NG52 or vehicle control. Cell lysates were subjected to immunoblotting with anti-K353la-PGK1 antibody for site-specific lactylation detection and anti-total PGK1 antibody for loading control for normalization. *n* = 3 per group. (D) Immunoblotting analysis of iNOS and PDH phosphorylation in M1-polarized THP1 and BMDMs treated with NG52 or vehicle control. *n* = 3 per group. (E) mRNA expression of *α-SMA*, *Col1α1*, *Fibronectin*, and *Timp1* was quantified in 3 murine models of liver fibrosis induced by CCl_4_ injection, MCD diet, or BDL with NG52 or vehicle control. *n* = 6 per group. (F) mRNA expression of *Tnf-α*, *Il-1β*, and *Il-10* was quantified in the mice. (G) Serum TNF-α, IL-1β, and IL-10 were measured by ELISA in the indicated groups. *n* = 6 per group. (H) The degree of fibrosis was evaluated by H&E, Masson’s trichrome, Sirius Red, collagen I, and α-SMA immunohistochemical staining, and the degree of inflammation was evaluated by Ly6G immunohistochemical staining in the indicated groups. Scale bars, 50 μm. (I) Protein expression of collagen I, α-SMA, and iNOs was analyzed by Western blot analysis in liver samples. Data were presented as mean ± SEM. Data were analyzed by an unpaired Student *t* test, with statistical significance set at *P < 0.05 and **P < 0.01.

To evaluate the functional role of PGK1 in fibrosis, we used the selective inhibitor NG52 (50 mg/kg/d). In vitro, NG52 attenuated M1 polarization and reduced PGK1 enzymatic activity in BMDMs (Fig. 7A and B), accompanied by decreased PGK1 K353 lactylation and suppressed mitochondrial translocation (Fig. 7C and D). In coculture, NG52-treated BMDMs suppressed HSC activation (Fig. S8D). In 3 murine fibrosis models, NG52 treatment down-regulated fibrosis-related genes (Fig. 7E) and proinflammatory cytokines while elevating anti-inflammatory IL-10 (Fig. 7F and G). Histological staining (hematoxylin–eosin [H&E], Masson’s trichrome, and Sirius Red) and immunohistochemistry for alpha-smooth muscle actin (α-SMA) and collagen I revealed reduced fibrosis, collagen deposition, and inflammatory infiltration (Fig. 7H and I). NG52 also directly inhibited HSC activation in primary cells from fibrotic mice (Fig. S8E and F). These results demonstrate that PGK1 inhibition attenuates hepatic fibrosis by impeding macrophage glycolytic reprogramming and HSC activation.

In summary, these results support the notion that the FSP1-mediated glycolysis/PGK1 lactylation positive feedback loop can “lock” the metabolic reprogramming of hepatic macrophages in fibrotic livers.

### Targeting PGK1-K353 lactylation with K353-pe ameliorates hepatic fibrosis by reducing inflammation, metabolic rewiring, and HSC activation

**Fig. 8.**
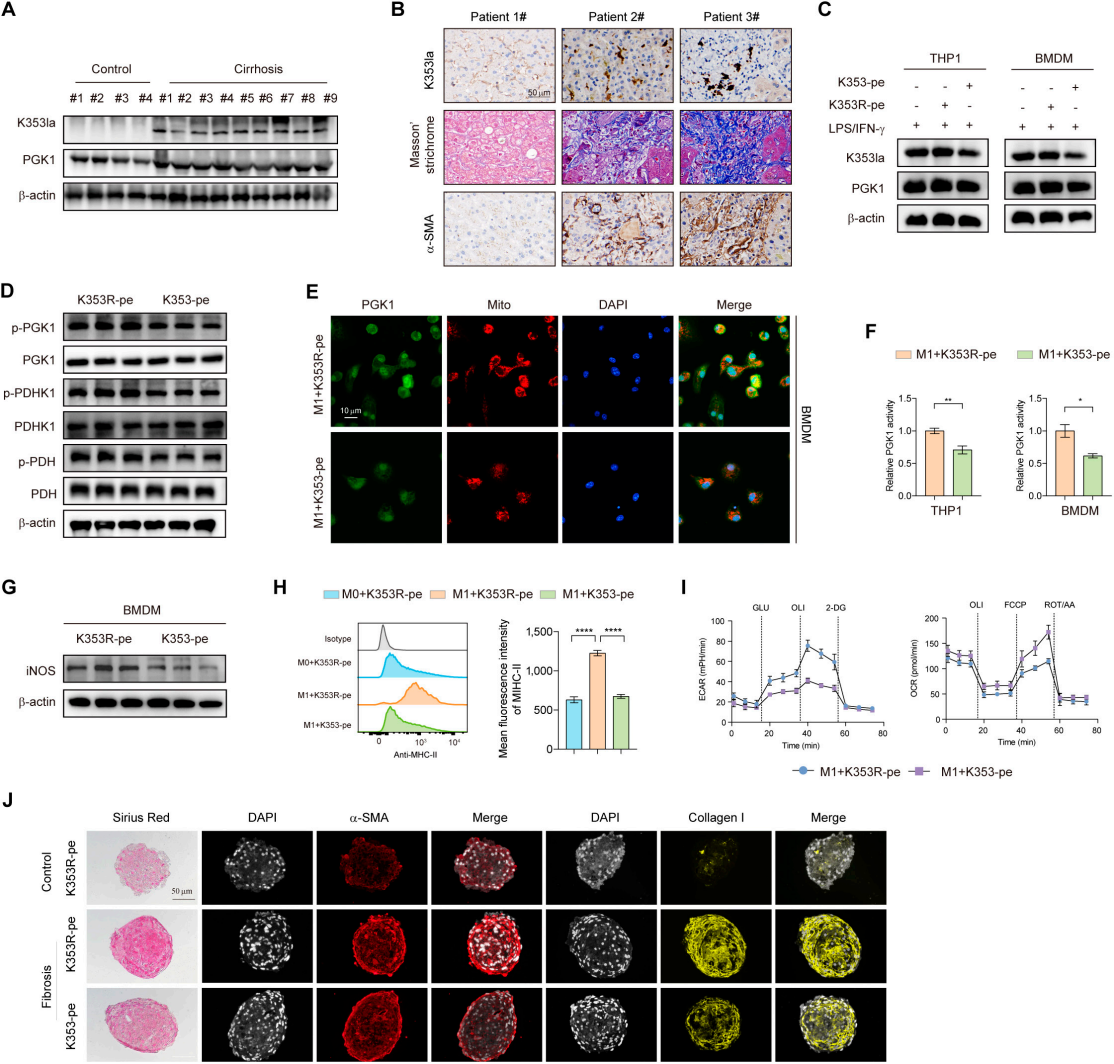
Inhibiting PGK1 K353 lactylation with a peptidic inhibitor disrupts the glycolysis/PGK1 lactylation positive feedback loop in FSP1^+^ macrophages. (A) Liver tissues were collected from 24 patients with liver cirrhosis and 12 patients undergoing hemangioma surgery for Western blotting analysis. (B) Clinical liver fibrosis tissues were subjected to immunohistochemistry staining, and the representative staining results of samples with PGK1 K353la levels were shown. Scale bar, 50 μm. *n* = 20 for cirrhosis samples and *n* = 8 for the controls. (C) PGK1 lactylation was analyzed in M1-polarized THP1 cells and BMDMs, followed by treatment with K353-peptide (50 μM) or K353R-peptide (50 μM) as control for an additional 24 h. Western blot analysis was performed to detect K353 lactylation and PGK1 expression. *n* = 3 biological replicates. (D) PGK1, PDHK1, and PDH phosphorylation was detected by immunoblotting analysis. *n* = 3 biological replicates. (E) M1-polarized BMDMs were treated with K353-peptide or K353R-peptide, followed by staining with anti-PGK1 antibody and MitoTracker. Nuclei were counterstained with DAPI. *n* = 3 biological replicates. (F) The enzymatic activity of PGK1 was assessed in the indicated groups. *n* = 3 biological replicates. (G) Immunoblotting analysis of iNOS was assessed in the indicated groups. *n* = 3 biological replicates. (H) The MFIs of MHC-II were detected in BMDMs subjected to the indicated treatments, as determined by flow cytometry. *n* = 3 biological replicates. (I) The ERCR and OCR were determined in M1 polarized-BMDMs receiving K353R-pe or K353-pe. *n* = 3 biological replicates. (J) Sirius Red staining and collagen I IF staining were used to assess fibrosis severity, while α-SMA IF staining evaluated HSC activation. Scale bars, 50 μm. *n* = 3 biological replicates. Data were presented as mean ± SEM. Statistical significance was determined by unpaired Student *t* test (E) or one-way ANOVA (H). *P < 0.05, **P < 0.01, ****P < 0.0001.

To assess PGK1-K353 lactylation (PGK1-K353la) in human liver disease, we analyzed cirrhotic and normal tissues. Western blot revealed comparable total PGK1 levels between groups, but robust PGK1-K353la expression specifically in cirrhotic livers (Fig. 8A). Immunohistochemical analysis of clinical fibrosis specimens confirmed a significant positive correlation between PGK1-K353la levels and both α-SMA and collagen I expression in fibrotic tissues (Fig. 8B). These findings establish PGK1-K353la as a critical mediator of fibrosis progression, with dual potential as a novel biomarker for fibrosis staging and a therapeutic target in cirrhotic liver diseases.

Considering that 2-DG and NG52 may broadly affect glycolysis or PGK1 function, we designed a CPP (K353-pe) incorporating a CPP motif based on sequence analysis surrounding the PGK1 K353 site to specifically target PGK1 K353 lactylation, along with a scrambled K353R-pe as a negative control (Fig. S9A and B). In M1-polarized macrophages, K353-pe treatment significantly reduced PGK1 lactylation (Fig. 8C and Fig. S9C), subsequently decreasing PGK1 phosphorylation, mitochondrial translocation (Fig. 8D and E and Fig. S9D), and PDHK1/PDH activation (Fig. 8D), while suppressing PGK1 enzymatic activity (Fig. 8F). This peptide markedly inhibited M1 polarization (Fig. 8G and H and Fig. S9E) and shifted metabolic flux from glycolysis (reduced ECAR) to oxidative phosphorylation (increased OCR, Fig. 8I). These effects collectively attenuated the activation of HSCs by macrophages (Fig. S9F and G), demonstrating that K353-pe disrupts the fibrogenic lactylation–glycolysis axis by specifically inhibiting PGK1 K353 lactylation. To evaluate the therapeutic potential of K353-pe against liver fibrosis, we established a physiologically relevant 3-dimensional (3D) liver fibrosis spheroid model. Using 3D NACs-origami technology, primary human hepatocytes, HSCs, liver endothelial cells, and Kupffer cells were assembled into spheroid structures. Peripheral blood mononuclear cells (PBMCs) from healthy donors were incorporated into the culture system, followed by TGF-β1-induced fibrosis and maintenance in a dynamic coculture environment mimicking hepatic microenvironment. K353-pe treatment significantly reduced collagen deposition and suppressed HSCs activation in this model (Fig. 8J). This positions PGK1-K353la as both a prognostic biomarker and a druggable target for metabolic intervention in liver fibrosis.

**Fig. 9.**
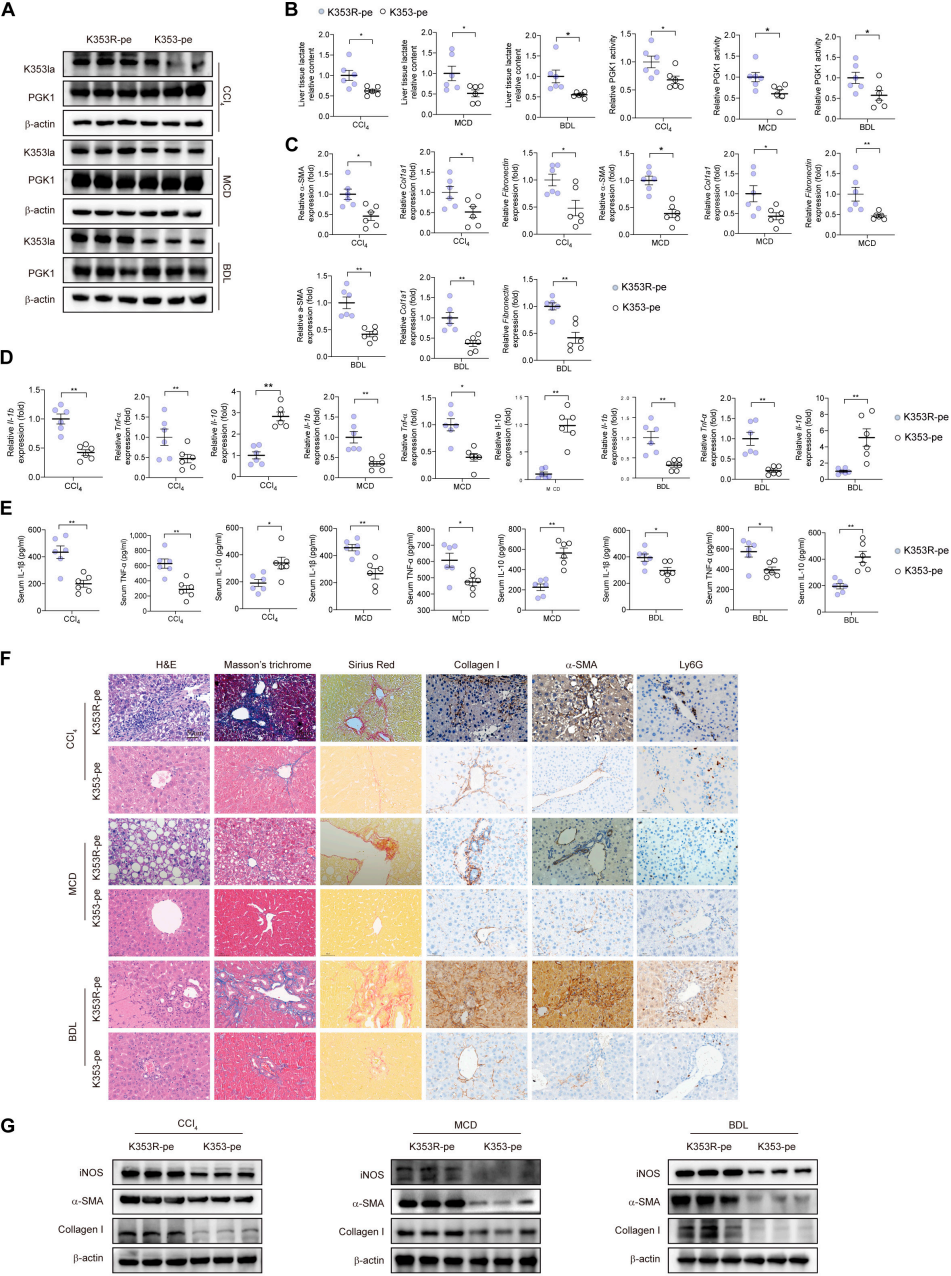
Targeting lactylation of PGK1 K353 via a peptidic inhibitor alleviates the severity of liver fibrosis. (A) PGK K353la was detected in fibrotic mice using 3 distinct models: CCl_4_ injection, MCD diet, and BDL. Mice were treated with either K353-peptide (K353-pe) or K353R-peptide (K353R-pe, control). *n* = 6 per group. (B) Liver tissue lactate and PGK1 activity were determined in fibrotic mice with the indicated treatment. *n* = 6 per group. (C) mRNA expression of *α-SMA*, *Col1a1*, and *Fibronectin* was quantified in the indicated groups. *n* = 6 biological replicates per group. (D) mRNA expression of *Tnf-α*, *Il-1β*, and *Il-10* was quantified in the indicated groups. *n* = 6 per group. (E) Serum TNF-α, IL-1β, and IL-10 were measured by ELISA in the indicated groups. *n* = 6 per group. (F) The degree of fibrosis was assessed by H&E, Masson’s trichrome, Sirius Red staining, collagen I, and α-SMA immunohistochemical staining, and the degree of inflammation was evaluated by Ly6G immunohistochemical staining in the indicated groups. *n* = 6 per group. Scale bars, 50 μm. (G) Protein expression of collagen I, α-SMA, and iNOS was analyzed in liver samples by Western blot analysis. *n* = 3 technical replicates per group. Data were presented as mean ± SEM; Statistical significance was determined by unpaired Student *t* test. *P < 0.05, **P < 0.01.

In vivo administration of K353-pe significantly reduced hepatic PGK1 lactylation (Fig. 9A), suppressed lactate production and inhibited PGK1 enzymatic activity (Fig. 9B) without observed toxicity (Fig. S10A). K353-pe significantly suppressed the expression of fibrosis-related genes in fibrotic liver (Fig. 9C). Additionally, K353-pe treatment down-regulated proinflammatory cytokines IL-1β and TNF-α while up-regulating the anti-inflammatory cytokine IL-10 (Fig. 9D). In accordance with these results, histopathological analyses (H&E, Masson’s trichrome, and Sirius Red staining) confirmed that K353-pe effectively attenuated hepatic fibrosis (Fig. 9E). Immunohistochemical staining and Western blot analyses further corroborated these findings, revealing reduced collagen deposition, neutrophil infiltration, and iNOS expression (Fig. 9E and F). Notably, K353-pe treatment also inhibited HSCs activation, as evidenced by decreased α-SMA expression in primary HSCs isolated from fibrotic livers (Fig. S10B).

Collectively, these findings demonstrate that the K353-pe, which specifically inhibits PGK1 lactylation at K353, effectively halts the progression of hepatic fibrosis.

## Discussion

This study identifies a novel mechanism by which FSP1^+^ macrophages drive liver fibrosis progression through a glycolysis-PGK1 lactylation self-reinforcing loop. FSP1 is up-regulated in M1 macrophages and correlates with fibrosis severity in human and murine livers. Myeloid-specific FSP1 knockout inhibits M1 polarization, reduces proinflammatory cytokine secretion, and attenuates immune cell infiltration, thereby ameliorating liver fibrosis. Mechanistically, FSP1 interacts with PKM2 to inhibit its ubiquitination-dependent degradation, enhancing glycolysis and establishing a glycolysis-PGK1 lactylation positive feedback loop that sustains macrophage pro-fibrotic phenotypes. PGK1 K353 lactylation acts as a molecular switch coordinating mitochondrial pyruvate metabolism and glycolytic reprogramming. A novel peptide inhibitor (K353-pe) targeting this modification disrupts the pathological circuit, offering a promising therapeutic strategy. Collectively, these findings establish PGK1 K353 lactylation as a central regulator of macrophage metabolic reprogramming and inflammatory polarization in fibrogenesis.

Uncontrolled inflammation drives the transition from self-limited tissue repair to persistent liver fibrosis. Macrophages, as key immune regulators in hepatic inflammation, orchestrate fibrosis progression or resolution [6,30]. Metabolic reprogramming and macrophage polarization form a bidirectional pathogenic axis in hepatic fibrosis pathogenesis [31,32]. Enhanced glycolysis not only provides energy substrates and metabolic intermediates for M1 macrophages activation but also generates metabolites that promote proinflammatory genes transcription through epigenetic modifications [33,34]. Activated M1 macrophages exacerbate liver injury through cytokine/reactive oxygen species (ROS) secretion and remodel the metabolic microenvironment to sustain glycolysis [35]. FSP1^+^ macrophages represent a proinflammatory subset that actively contributes to fibrogenesis. Elevated FSP1 expression in macrophages from both human and murine fibrotic livers was correlated with fibrosis severity. Myeloid-specific FSP1 knockout reduced neutrophil infiltration, suppressed M1 polarization, and attenuated hepatic inflammation and fibrosis, which position FSP1 as a multifaceted regulator of macrophage-driven fibrogenesis. We unveil a novel mechanism in which FSP1 directly binds to the bifunctional enzyme PKM2, integrating metabolic regulation with proinflammatory reprogramming. Upon LPS/IFN-γ stimulation, PKM2 up-regulation drives proinflammatory factor IL-1β production via promoter binding during glycolysis [36]. We further demonstrate that FSP1 stabilizes PKM2 by inhibiting ubiquitination, thereby enhancing PKM2-mediated M1 polarization and glycolysis in activated macrophages. This FSP1–PKM2 interaction amplifies lactate accumulation and pro-fibrotic macrophage phenotypes. Together, these findings identify FSP1 as a metabolic–immune checkpoint regulating macrophage-driven fibrogenesis.

In pathological contexts such as the tumor microenvironments or chronic liver diseases, aberrant glycolysis drives excessive lactate production [17]. Accumulated lactate acts as a critical metabolite and signaling molecule, modifying proteins through both enzymatic (e.g., KAT2B-mediated) and nonenzymatic pathways, while also fueling the TCA cycle via LDH-dependent conversion to pyruvate [26,37]. Nonhistone lactylation has emerged as a pivotal epigenetic mechanism [38], regulating protein function by altering lysine residues to influence conformation [39], charge distribution [40], and subcellular localization [41]. This dynamic PTM modulates key pathological processes including metabolic reprogramming [38], DNA repair [26,37], and immune regulation [42]. A critical discovery of this study is the identification of PGK1 K353 lactylation as a molecular switch governing macrophage metabolic fate. Metabolic reprogramming in M1 polarized macrophages involves a shift from mitochondrial pyruvate metabolism and oxidative phosphorylation to glycolysis [43]. During M1 polarization, lactate accumulation drives KAT2B-mediated lactylation of PGK1 at K353, enhancing its glycolytic activity and promoting interaction with PDHK1. Activated PDHK1 inhibits PDH, diverting pyruvate from mitochondrial oxidation to sustain cytoplasmic glycolysis. This creates a self-reinforcing loop where glycolysis promotes lactylation, which in turn amplifies glycolytic flux. Notably, this mechanism is disrupted by FSP1 knockdown, highlighting its dependence on the FSP1–PKM2 axis.

To address PGK1-K353 lactylation modification, we designed a targeted peptide that specifically avoids nonspecific interference with other lactylation sites or proteins, thereby reducing off-target effects. This precision strategy opens a new direction for liver fibrosis treatment. Elevated PGK1 K353 lactylation in liver tissues from cirrhosis patients, which shows a correlation with the fibrosis stage, strongly emphasizes the translational potential and clinical relevance of this biological pathway. To target this mechanism, we developed CPP K353-pe, which specifically inhibits PGK1 lactylation. In preclinical models, K353-pe suppresses M1 polarization, reduces glycolytic activity, and alleviates hepatic fibrosis without apparent toxicity. This peptide-based strategy offers a precision therapy compared to broad-spectrum metabolic inhibitors, addressing the unmet need for targeted therapies in fibrotic liver diseases. Our study adds to the growing literatures on lactylation as an epigenetic regulator of immune cell function. By linking FSP1-driven glycolysis to PGK1 lactylation, we demonstrate how metabolic intermediates can orchestrate PTMs to sustain pro-fibrotic phenotypes. This mechanism may extend to other inflammatory contexts, such as cancer and autoimmune diseases, where lactate-driven lactylation has been implicated in immune cell polarization.

In summary, this study defines a critical role for the FSP1/PKM2/glycolysis/PGK1 lactylation axis in hepatic fibrosis. By uncovering a metabolic–epigenetic feedback loop in macrophages, we provide mechanistic insights into how chronic inflammation drives fibrogenesis. The development of K353-pe as a targeted inhibitor of this pathway highlights potential therapeutic strategies to disrupt the vicious cycle of metabolic reprogramming and inflammation in liver diseases.

## Materials and Methods

### Human samples

The utilization of human samples was approved by the Medical Ethics Committee of Tongji Hospital Affiliated to Tongji University (approval no. 2023-008). Written informed consent was obtained from each participant in accordance with the Declaration of Helsinki. This study utilized human liver samples acquired during hepatectomy or biopsy. A total of 12 liver samples from patients with benign liver diseases (from hemangioma patients without fibrosis, steatosis, diabetes, alcoholism, or viral hepatitis, including 12 liquid nitrogen-frozen samples and 8 paraffin-embedded samples) and 24 liver samples from patients with histologically confirmed cirrhotic liver (including 24 liquid nitrogen-frozen samples and 20 paraffin-embedded sections) were collected. Control individuals had no history of diabetes, alcoholism, or viral hepatitis, and their control liver samples were obtained from normal liver tissues at the resection margin of hemangiomas. Diagnosis of liver fibrosis was confirmed pathologically by senior pathologists based on H&E staining and Sirius Red staining results. To detect serum FSP1 levels, peripheral blood was obtained from 62 cirrhotic patients and 16 healthy blood donors.

### Mice

All experimental animals were housed in a specific pathogen-free animal facility under a 12-h light/dark cycle, with temperature maintained at 23 ± 2 °C and relative humidity at 40% to 70%. Unless otherwise specified, all experiments used 6- to 8-week-old sexually mature mice. Mice in all groups received food and water ad libitum following random group assignment. All handling and experiments complied with protocols approved by Tongji Hospital’s Animal Ethics Committee (approval no. 20230101-DW-026).

Three liver fibrosis models were used in this study. CCl_4_-induced fibrosis model: 6- to 8-week-old male mice were randomly divided into 2 groups receiving intraperitoneal injection of olive oil or CCl_4_ (0.5 ml/kg body weight), 3 times a week for 4 weeks; MCD diet-induced fibrosis model: 6- to 8-week-old male mice were fed MCD (TP 3005G, Trophic Animal Feed High-tech, China) or MCS diet for the control (TP3005GS, Trophic Animal Feed High-tech, China) for 4 weeks; BDL-induced fibrosis model: Male mice of the same age underwent surgery under isoflurane inhalation anesthesia. After abdominal skin disinfection, a midline incision was made to expose the common bile duct, which was ligated with 5-0 nonabsorbable sutures. Tissue samples were harvested 14 days postoperatively.

Male WT C57BL/6J mice were obtained from SLAC Laboratory Animal Co., Ltd., and *Fsp1^f/f^* and *LysM-cre* mice were procured from Cyagen Biosciences. For myeloid-specific knockout, *LysM^cre^Fsp1^f/f^* mice were produced by breeding *Fsp1^f/f^* mice with *LysM-cre* mice. The experimental protocol received approval from the Animal Ethics Committee of Tongji Hospital Affiliated to Tongji University.

### Enzyme-linked immunosorbent assay

Serum concentrations of FSP1 protein and secreted cytokines (IL-1β, IL-10, and TNF-α) were measured using enzyme-linked immunosorbent assay (ELISA) kits (biotechwell, China) according to the manufacturer’s instructions. Samples (100 μl each) were transferred to antibody-coated 96-well microplates and incubated (37 °C, 2 h). Postincubation, wells were washed extensively with buffer. Following incubation, plates were washed thoroughly with assay buffer. Biotin-conjugated detection antibodies (100 μl/well) were then added and incubated at 37 °C for 1 h. After subsequent washing, plates were incubated with streptavidin–horseradish peroxidase (HRP) conjugate (100 μl/well) at 37 °C for 30 min. Following wash steps, TMB substrate (100 μl per well) was added to each well for color development. After incubation in darkness for 15 min at room temperature, the reaction was terminated with stop solution. Absorbance was immediately measured at 450 nm using a microplate reader. Cytokine concentrations were calculated based on standard curves derived from recombinant protein reference standards.

Serum levels of alanine aminotransferase (ALT, C009-1, Nanjing Jiancheng, China) and aspartate aminotransferase (AST, C010-2, Nanjing Jiancheng, China) were detected using kits. Following detection, samples were analyzed using a fully automated biochemical analysis system (Thermo Fisher Scientific SkanIt Software 6.1.1.7).

### Histopathology and immunohistochemistry

Mouse liver samples underwent 10% formalin fixation and paraffin embedding. Sections (5 μm thick) were cut using a microtome, mounted on slides, deparaffinized in xylene (2 × 10 min), and rehydrated through graded ethanols (100%, 95%, 85%, and 75% for 5 min each). To quench endogenous peroxidase activity, tissue sections were incubated in hydrogen peroxide (H_2_O_2_) solution at ambient temperature for 10 min, followed by heat-induced antigen-retrieval in citrate buffer, pH 6.0. Nonspecific protein interactions were minimized by applying 5% normal goat serum (C0265, Beyotime, China) for 30 min under room temperature conditions. Following aspiration of the blocking solution, primary antibodies were applied and incubated at 4 °C overnight in a humidified chamber to maintain hydration. Sections underwent 3 phosphate-buffered saline (PBS) washes (5 min each) before 30-min room temperature incubation with species-specific secondary antibodies conjugated to either fluorescent probes or HRP. For chromogenic detection, sections were incubated with 3,3′-diaminobenzidine (DAB, DAB2031, MXB biotechnologies, China) substrate solution until optimal brown precipitation developed. Nuclei were counterstained with Mayer’s hematoxylin (2 min) prior to dehydration.

For Sirius Red staining, sections were immersed in picric acid–Sirius Red staining solution at room temperature for 1 h, rinsed twice with 0.5% acetic acid solution (5 min each), and dehydrated with absolute ethanol. Five random fields of view (200× or 400× magnification) were selected from each section, and the positive area of Sirius Red staining was quantified by analyzing the stained area using NIH ImageJ software.

For Masson’s trichrome staining (GP1032, Servicebio, China), sections were fixed in Bouin’s solution (60 °C, 1 h) post-deparaffinization/rehydration. Nuclear staining employed Weigert’s iron hematoxylin (5 min), followed by cytoplasmic staining with Biebrich scarlet-acid fuchsin (15 min). Contrast was enhanced via phosphomolybdic–phosphotungstic acid differentiation (15 min) before collagen-specific aniline blue staining (10 min). Final processing included 5% acetic acid exposure (5 min), graded ethanol dehydration, and neutral gum mounting.

### RNA isolation and qRT-PCR assays

Total RNA was extracted from tissue samples or cells using TRIzol reagent (15596-026, Invitrogen, USA), purified by isopropanol precipitation, and quantified by NanoDrop UV spectrophotometry to assess concentration and purity via the A260/A280 ratio. For reverse transcription, 1 μg of total RNA was used with the HiScript III RT SuperMix kit (Vazyme R222, Vazyme Biotech, China) to synthesize complementary DNA (cDNA). Real-time (RT) quantitative PCR was performed using the ChamQ Universal SYBR qPCR Master Mix kit (Vazyme q711, Vazyme Biotech, China) on a QuantStudio 5 real-time PCR system (Thermo Fisher), with β-actin as the internal reference gene. Relative gene expression was calculated using the 2^−ΔΔCt^ method. This calculation inherently results in setting control sample normally to 1, establishing it as the baseline. All experimental group data are expressed as fold change relative to this calibrator. Data from biological replicates are presented as the mean fold change ± the standard error of the mean (SEM). All primers were validated for specificity via BLAST alignment, with sequences detailed in STAR Methods. Primers were synthesized by Sangon Biotech (Shanghai, China).

### Immunoblotting

Cells were collected and lysed on ice for 1 h using prechilled radioimmunoprecipitation assay (RIPA) lysis buffer with protease inhibitor. The supernatant was centrifuged (13,000×*g*, 10 min, 4 °C) for protein collection. Proteins underwent sodium dodecyl sulfate–polyacrylamide gel electrophoresis (SDS-PAGE) separation (stacking gel: 80 V; resolving gel: 120 V) and wet transfer onto 0.45-μm PVDF membranes (300 mA, 90 min). After 1-h blocking with 5% TBST-skim milk at room temperature, membranes were incubated overnight at 4 °C with shaking in primary antibodies (manufacturer-specified dilutions). Following TBST washes, HRP-conjugated secondary antibodies (1:5,000) were applied for 1 h at RT Protein. Protein bands were visualized by enhanced chemiluminescence (ECL, AP34L035, HeYuan Bio, China). β-Actin served as the loading control to verify loading consistency via repeated experiments, ensuring result reliability. Antibody information used in this study is detailed in STAR Methods, and all antibodies were validated for specificity by Western blot.

### Immunofluorescence and confocal microscopy

Liver tissue sections (5 μm; formalin-fixed, paraffin-embedded) underwent deparaffinization, graded hydration, and antigen retrieval. In vitro cultured cells on coverslips were fixed with 4% paraformaldehyde and permeabilized with 0.1% Triton X-100. All samples were blocked with normal goat serum to minimize nonspecific binding. Primary antibodies (vendor-recommended dilutions) were incubated in a humidified chamber overnight at 4 °C. After PBST washing, Alexa Fluor 488/594-conjugated secondary antibodies were applied. Nuclei were stained with 4′,6-diamidino-2-phenylindole (DAPI), and samples mounted in antifade medium (G1401, Servicebio, China). Images were captured using a Nikon confocal laser scanning microscope (Nikon, Japan). For quantitative analysis, ImageJ was used to measure mean fluorescence intensity in at least 3 random fields and calculate the percentage of positive cells. Isotype control antibodies were included throughout the experiment to exclude nonspecific staining, with consistent exposure parameters maintained across groups.

### Primary cell isolation and culture

Femurs/tibiae from 6- to 8-week-old male mice were aseptically harvested. Marrow was flushed with Dulbecco's Modified Eagle Medium (DMEM) via 20-ml syringe/30 G needle. After red blood cell lysis (C3702, Beyotime, China), cells were washed with PBS and resuspended in DMEM containing 20 ng/ml M-CSF (315-02-100μg, PeproTech, USA) and 10% fetal bovine serum (FBS, 11965092, GIBCO, USA). Medium was replaced on day 4, and fully differentiated BMDMs were obtained on day 7. Lentivirus expressing FSP1 and vector were used to transfect BMDMs for 48 h. Cells were then treated with 100 ng/ml LPS (HY-D1056, MCE, USA) and 20 ng/ml IFN-γ (HY-P7071, MCE, USA) for 24 h.

Liver macrophages were isolated by portal vein perfusion. The liver was perfused with prewarmed (37 °C) 0.05% collagenase type IV (dissolved in Ca^2+^/Mg^2+^-containing HBSS), filtered through a 70-μm nylon mesh (Falcon), and cells were collected by low-speed centrifugation at 50×*g*. Primary hepatocytes were isolated by 50% Percoll density gradient centrifugation (200×*g*, 20 min) and seeded in DMEM with 10% FBS. To enrich liver macrophages, nonparenchymal cells (NPCs) resuspended in HBSS were layered onto a 25% to 50% Percoll gradient (P8370, Solarbio, China) and centrifuged at 1,800×*g* for 15 min. Cells from the interphase were collected, seeded in plates, and incubated at 37 °C for 20 min to allow macrophage adhesion; nonadherent cells were removed by medium replacement.

For primary HSC isolation, livers from 4 to 5 mice were perfused in situ via the portal vein with a mixed enzyme solution containing Pronase (P5147, Sigma-Aldrich, USA) and 0.05% collagenase type IV (C5138, Sigma-Aldrich, USA). After harvest, livers were mechanically minced, filtered through a 70-μm cell strainer and collected to generate single-cell suspensions. NPCs in the supernatant were obtained by low-speed centrifugation at 50×*g* for 5 min, followed by centrifugation at 700×*g* for 10 min to enrich cell pellets. Pellets were washed twice with Gey’s balanced salt solution (GBSS/B buffer), then subjected to Nycodenz density gradient centrifugation (AN1002424, Accurate Chemical, USA) at 1,380×*g* for 17 min with natural deceleration. HSC purity was validated by detecting autofluorescence of intracellular vitamin A lipid droplets using a Zeiss LSM 700 confocal laser scanning microscope.

### BMDMs coculture with HSCs

BMDMs from different treatment groups were cocultured with primary HSCs at a 1:1 ratio in DMEM medium containing 10% FBS. The coculture system was established in 6-well plates, with HSCs seeded in the lower chamber and BMDMs plated on Transwell culture inserts (BioFil) with a 0.4-μm pore size.

### Cell culture

The human monocytic leukemia cell line THP1 was cultured in THP1-specific medium (CM-0233, Pricella, China) and maintained in a humidified incubator at 37 °C with 5% CO₂. THP1 monocytes were induced to differentiate into macrophages by incubation with 150 nM phorbol 12-myristate 13-acetate (PMA, HY-18739, MCE, USA) for 24 h, followed by an additional 24 h culture in fresh medium. M1 polarization of macrophages was achieved by incubation with 100 ng/ml LPS and 20 ng/ml IFN-γ.

### DNA transfection, virus packaging, and lentiviral infection

A total of 24 expression plasmid vectors were constructed and used in this study. Transfection experiments for HEK293T cells were performed using Lipofectamine 8000 reagent (C0533, Beyotime, China). Cells were seeded into 10-cm dishes (1 × 10^7^ cells/well) and cultured for 24 h before transfection. The transfection system for each well contained 15 μg of plasmid and 24 μl of Lipofectamine 8000 reagent.

WT and K353R mutant PGK1 cDNAs were cloned into pLVX-CMV-Flag-Puro lentiviral vectors. For lentiviral production, HEK293T cells underwent Lipofectamine 3000 (L3000015, Thermo Fisher Scientific, USA)-mediated cotransfection with lentiviral constructs and packaging plasmids (pMD2.G/psPAX2). Viral supernatants were harvested at 48 and 72 h posttransfection, then concentrated. Bone marrow (BM) precursor cells were transduced with lentivirus in polybrene-supplemented medium (8 μg/ml final concentration) and then differentiated into BMDMs using the differentiation medium.

### RNA interference

To achieve specific gene silencing, this study employed synthesized target-specific siRNAs and synthesized negative control siRNAs. For 12-well plates, 24 pmol of siRNA and 3 μl of Rfect V2 siRNA Transfection Reagent (11041, Biodragon, China) were separately diluted in 50 μl of transEnhancer (11041, Biodragon, China), incubated at 37 °C for 5 min for precomplexation. The 2 mixtures were then combined in equal volumes and incubated at 37 °C for an additional 15 min to form siRNA–liposome complexes, which were subsequently added to the BMDM culture. For THP1, RFect^SP^ siRNA Transfection Reagent (11024, Biodragon, China) was used according to the manufacturer’s protocol. Gene silencing efficiency was validated 48 h posttransfection via RT-qPCR or immunoblotting. Functional experiments were performed only after confirming effective silencing. A nontargeting negative control siRNA was used in all experiments. Detailed siRNA sequences are provided in STAR Methods.

### Flow cytometry

To assess M1 macrophage polarization, BMDMs were first incubated with anti-mouse FITC MHC class II antibody (FITC-65122, Proteintech, China) for 30 min at room temperature in the dark. After PBS washing 3 times, cells were resuspended in 200 μl of PBS for analysis on a BD FACS Celesta. Appropriate fluorescence compensation was applied during data acquisition, and gating strategies were established using FlowJo software to calculate phenotypic proportions.

### RNA sequencing and data analysis

Total RNA was extracted from BMDMs of the LPS/IFN-γ-induced group (M1+siCtrl group) and LPS/IFN-γ-induced FSP1-knockdown group (M1+si*FSP1* group) using a modified TRIzol method. Sequencing services were performed by APExBIO using the BGISEQ500 high-throughput sequencing platform with paired-end 150-bp reads. Differentially expressed genes were screened based on the criteria: fold change ≥1.5 and *P* value < 0.05. Sequencing data were integrated and analyzed via the Oebiotech online analysis platform.

### Multiplex immunohistochemistry

This study employed multiplex fluorescence immunohistochemistry for staining analysis of 5-μm-thick formalin-fixed, paraffin-embedded whole tissue sections. After deparaffinization, sections underwent multiple rounds of primary antibody incubation and tyramide signal amplification (TSA) labeling. Taking FSP1 antibody staining (ab197896, Abcam, Cambridge, UK) as an example, sections were incubated with anti-FSP1 primary antibody at 4 °C for overnight, followed by incubation with rabbit anti-IgG HRP-conjugated secondary antibody (A0216, Beyotime, China) for 30 min. After each staining round, sections were thoroughly washed with buffer, transferred to preheated (90 °C) citrate antigen retrieval solution, subjected to heat-mediated antigen retrieval at 20% maximum power for 15 min, then allowed to cool to room temperature naturally.

Following the above protocol, staining for the following targets was sequentially performed: anti-Albumin receptor antibody (16475-1-AP, Proteintech, China) coupled with TSA 670, anti-F4/80 antibody (70076, Cell Signaling Technology, USA) coupled with TSA 520, and anti-CD31 antibody (77699, Cell Signaling Technology, USA) coupled with TSA 440. Stringent washing was performed after each staining round to eliminate cross-reactivity. Upon completion of all target staining, sections were counterstained with DAPI. Two drops of DAPI working solution were applied to each section, followed by distilled water washing and mounting with antifade mounting medium. After air-drying, sections were subjected to multichannel fluorescence imaging using an Aperio Versa 8 whole-slide scanning system (Leica).

### Proteomics analysis

Total proteins were extracted from mouse liver tissues, digested with trypsin, and labeled using an iTRAQ labeling kit (4381663, AB SCIEX, USA). Labeled samples were subjected to quantitative analysis by liquid chromatography–tandem mass spectrometry (LC-MS/MS). Proteomics detection and iTRAQ relative quantification services were provided by PTM-Biolab (China). Biological function classification of differentially expressed proteins was performed via Gene Ontology (GO) functional annotation and KEGG pathway enrichment analysis to decipher their involvement in core biological processes and signaling pathways.

### Lactic acid concentration assay

For intracellular lactic acid concentration measurement, BMDMs or THP1-derived macrophages underwent PBS rinsing prior to lysis using the specified buffer from the L-Lactic Acid Detection Kit (BC2235, Solarbio, China). Postcentrifugation (12,000×*g*, 15 min, 4 °C), supernatants were harvested. Lactic acid levels were quantified per kit specifications, while total protein concentration was determined with a BCA Protein Assay Kit (ZJ101, Epizyme Biotech, China). Finally, lactic acid concentration was normalized to milligrams of total protein, following the manufacturer’s specified protocol.

For lactic acid concentration measurement in liver tissues, equal amounts of liver tissue (20 to 50 mg) were precisely weighed, added to the lysis buffer of the L-Lactic Acid Detection Kit (BC2235, Solarbio, China), and fully lysed by mechanical homogenization. The homogenates were centrifuged at 12,000×*g* for 15 min at 4 °C, and the supernatants were used to determine lactic acid concentration according to the kit instructions. Each group was set with 3 to 5 biological replicates, and the final results were expressed as μmol/g wet weight tissue. All procedures were strictly performed following the kit instructions, and blank controls were set to correct background signals.

### LDH activity assay

LDH activity in liver tissues and cells was measured using a kit (BC0685, Solarbio, China), with all procedures strictly following the kit instructions.

### ECAR and OCR

Measurements were conducted with the Agilent Seahorse XF Analyzer according to the manufacturer’s instructions. For OCR measurement, 3 × 10^4^ cells were seeded into a Seahorse XF96 microplate and incubated overnight in XF Base Medium (Agilent). Mitochondrial function was assessed using the Agilent Seahorse XF Cell Mito Stress Test Kit (Agilent). OCR was measured by sequential addition of oligomycin (2.5 μM), carbonyl cyanide 4-(trifluoromethoxy) phenylhydrazone (2 μM), and rotenone (0.5 μM) + antimycin A (0.5 μM). For ECAR measurement, 3 × 10^4^ cells were seeded into a Seahorse XF96 microplate and incubated overnight. Glycolytic capacity was analyzed using the Agilent Seahorse XFp Glycolysis Rate Assay Kit (Agilent). ECAR was determined by sequential administration of glucose (10 mM) and rotenone (1 μM), followed by 2-DG (50 mM). Data were normalized to cell number.

### Co-IP assay

A total of 2 × 10^7^ cells were harvested and resuspended in prechilled PBS at 4 °C. After 3 washes, cells were lysed with 1 ml of RIPA buffer. The lysate underwent centrifugation (13,000 rpm, 30 min, 4 °C) with subsequent supernatant collection. For Co-IP, 50 μl of the protein lysate served as input control. The experimental group contained 950 μl of protein lysate, 40 μl of protein A/G agarose beads, and 5 μg of anti-PGK1 antibody or anti-Flag magnetic beads (P2202M, Beyotime, China) or anti-HA magnetic beads (P2206M, Beyotime, China). The IgG control group included 950 μl of protein lysate, 40 μl of protein A/G agarose beads (P2197M, Beyotime, China), and 5 μg of IgG. Experimental and IgG-control samples were subjected to shaking incubation overnight at 4 °C. Subsequently, samples were centrifuged at 1,000 rpm for 5 min at 4 °C, and the supernatant was discarded. Protein A/G agarose beads received 3 washes with lysis buffer. The pellet was mixed with 40 μl of 2×SDS loading buffer and incubated at 99 °C for 10 min. Anti-Flag or anti-HA magnetic beads were mixed with 40 μl of 3× Flag Peptide or HA Peptide incubated in an ice bath and shaken for 1 h. Then, the supernatant was collected by centrifugation at 6,000×*g*, 4 °C for 30 s and stored for further use. Finally, samples were separated by SDS-PAGE for subsequent analysis.

### Molecular dynamics simulation methodology

All-atom molecular dynamics (MD) simulations were performed for 100 ns using GROMACS 2020.6. Protein topologies were generated with the AMBER99SB-ILDN force field. For the modified lysine residue (LYS), nonstandard residue topologies were created using Amber20 in conjunction with the GAFF force field to accurately parameterize the charges and bonding parameters of the chemical modification. The protein complex was then centered in a cubic TIP3P water box with an edge length of 10 nm, and an appropriate number of Na^+^ and Cl^−^ ions were added to neutralize the system.

Energy minimization was conducted in 2 stages: first, steepest descent minimization for 2,500 steps to rapidly resolve steric clashes, followed by conjugate gradient minimization for another 2,500 steps until the maximum force was below 1,000 kJ mol^−1^ nm^−1^. The system was then heated to 298.15 K over 100 ps under the NVT ensemble. Subsequently, a 100-ps equilibration under the NPT ensemble at 101.325 kPa was performed to allow the system density and box parameters to stabilize.

The production MD simulation was carried out for 100 ns under the NPT ensemble with periodic boundary conditions. Long-range electrostatic interactions were treated using the Particle Mesh Ewald method, with a cutoff of 1.0 nm. Van der Waals interactions were also truncated at 1.0 nm. The coupling time constant was set to 2 ps, and the integration time step was 2 fs. Trajectory frames were saved every 10 ps.

Following the simulation, the final 20 ns of the stable trajectory were extracted for thermodynamic and structural analyses. The binding free energy was calculated using the MM/PBSA method with the gmx_mmpbsa tool to decompose the energetic contributions to ligand binding affinity. Structural stability was monitored throughout the simulation via RMSD, radius of gyration (*R*_g_), and solvent-accessible surface area. To identify flexible regions, the RMSF was calculated for each residue. Analyses of hydrogen bond occupancy and interfacial water bridges were performed to characterize key polar interactions at the binding interface. All trajectory visualizations and analyses of conformational changes and residue movements at the binding site were conducted using PyMOL 2.4.1.

### 2-DG treatment in vivo

To systematically evaluate the intervention effects of glycolysis inhibition on liver fibrosis with different etiologies, this study employed 3 classical models for mechanism validation. In the CCl_4_- and MCD-induced liver fibrosis models, 2-DG (1,000 mg/kg, HY-13966, MCE, USA) was intraperitoneally injected every 3 days starting from the end of the second week until the end of the fourth week. Animals were sacrificed 48 h after the last administration, and specimens were collected. In the BDL surgical model, 2-DG (1,000 mg/kg) was intraperitoneally administered every 3 days starting from the seventh day postsurgery. All 2-DG solutions were freshly prepared in sterile saline. Specimen collection strictly adhered to animal ethics guidelines, and animals were deeply anesthetized before sacrifice to minimize suffering.

### Gene expression profile data

Gene expression datasets analyzed in this study were retrieved from the National Center for Biotechnology Information GEO (https://www.ncbi.nlm.nih.gov/geo/). The GSE55747 dataset comprises RNA-seq data from liver tissues of WT and FSP1 transgenic mice featuring proliferative knockout in a CCl_4_-induced liver fibrosis model, providing important omics research resources for exploring the molecular regulatory mechanisms of the FSP1 gene in the development of liver fibrosis. Additionally, the GSE84044 and GSE130970 datasets were included to investigate the correlation between FSP1 and human liver fibrosis. Data from these datasets were normalized and subsequently subjected to further analysis using Rstudio (Version: v.1.4.1106). DEGs between liver samples were detected using the GEO2R tool (https://www.ncbi.nlm.nih.gov/geo/geo2r). Only genes with an adjusted *P* value < 0.05 were retained as DEGs. Subsequently, GO plots were generated using the “ggplot2” R package [44]. KEGG pathway enrichment analysis for the identified DEGs was conducted using an online web tool (https://www.omicshare.com/tools/home/report/koenrich.html), which enabled the exploration of the biological pathways associated with the differentially expressed genes.

### Protein–protein interaction network construction

To evaluate the interaction between FSP1 and metabolic pathways, a protein–protein interaction network was generated using the Search Tool for the Retrieval of Interacting Genes (STRING, https://string-db.org/). The network was constructed with a minimum confidence threshold (combined score ≥ 0.4), and interaction pairs of differentially expressed genes were extracted from the STRING database. To further assess the target genes of FSP1, this study analyzed the correlation between FSP1 and glycolysis-related genes according to connectivity.

### Enrichment of lactylated modified peptides by immunoprecipitation

Peptides were dissolved in immunoprecipitation (IP) buffer (100 mM NaCl, 1 mM EDTA, 50 mM Tris-HCl, and 0.5% NP-40, pH 8.0). The supernatant was then transferred to prewashed lactylation antibody beads (PTM-1401RM, PTM Biolab, China), followed by gentle incubation on a rotary shaker at 4 °C. After incubation, the beads were washed 4 times with NETN buffer. Lactylated peptides bound to the beads were eluted 3 times with 0.1% trifluoroacetic acid elution buffer. Eluates were collected, vacuum freeze-dried, and desalted using C18 ZipTip columns according to the manufacturer’s protocol. The desalted samples were vacuum freeze-dried again prior to LC-MS analysis.

### LC-MS analysis

Peptides were dissolved and separated using a NanoElute ultrahigh-performance liquid chromatography system (Bruker, Karlsruhe, Germany). Analytes were ionized through a capillary ion source and followed by analysis with a timsTOF Pro mass spectrometer (Bruker). The parent ions and secondary fragments of peptides were detected and analyzed by a high-resolution time-of-flight (TOF) detector. Data acquisition was performed using the parallel accumulation-serial fragmentation (PASEF) methodology.

### PGK1 activity assay

3-PGK1 catalyzes the reaction between 3-phosphoglycerate (3-PG) and ATP to produce 1,3-bisphosphoglycerate (1,3-BPG). Subsequently, 1,3-BPG is converted to 3-phosphoglyceraldehyde and NAD^+^ by glyceraldehyde-3-phosphate dehydrogenase in the presence of nicotinamide adenine dinucleotide (NADH). By measuring the decrease in NADH concentration, the activity of PGK1 can be quantitatively determined according to the manufacturer’s instructions (G0885W, Grace Biotechnology, China).

### In vitro lactylation assay

Glutathione *S*-transferase-fused PGK1 protein was coincubated with HA-tagged KAT2B protein purified from HEK293T cells in reaction buffer (50 mM HEPES, pH 7.8, 30 mM KCl, 0.25 mM EDTA, 5.0 mM MgCl₂, 5.0 mM sodium butyrate, and 2.5 mM dithiothreitol), supplemented with 20 mM lactyl-CoA (HY-141540, MCE, USA) [26]. The reaction mixture was incubated at 30 °C for 30 min. 5× SDS loading buffer was added, and proteins were denatured by heating at 100 °C for 5 min in a boiling water bath. After separation by SDS-PAGE, samples were subjected to Western blot analysis using specified antibodies to assess the lactylation modification level of target proteins.

### NG52 treatment in vivo

To investigate the interventional effect of a PGK1 inhibitor on liver fibrosis, this study employed 3 classic models for mechanism validation. In CCl_4_ injection- and MCD diet-induced liver fibrosis models, animals were administered 50 mg/kg NG52 (dissolved in 200 μl of specific solvent, N413510, Aladdin, China) daily by gavage starting from the end of the second week until the end of the fourth week. For BDL model, NG52 intervention at the same dose and administration route was initiated on day 7 postsurgery and continued until day 14 postsurgery. All animals were euthanized 48 h after the last administration, and serum, liver, and other tissue specimens were systematically collected for subsequent pathological and molecular biological assays. Control groups received an equal volume of vehicle via the same route simultaneously.

### 3D liver spheroid construction

Human primary hepatocytes, HSCs, liver endothelial cells, and Kupffer cells (3,000 cells/well) were assembled into 3D liver spheroids in Corning 96-well plates using 3D NACs-origami technology (Puheng Biotech) [45]. Spheroids were precultured in DMEM/10% FBS for 24 h, followed by coseeding of allogeneic human PBMCs (7,500 cells/well) and additional 24 h coculture. Fibrosis was induced with TGF-β1 (5 ng/ml), with continuous culture maintained for 9 days. Therapeutic peptides were introduced on day 5, with coculture sustained until day 9.

### Peptide synthesis and preparation

All peptides were synthesized by Guoping Pharmaceuticals (Hefei, China). Following synthesis, peptides were purified by high-performance liquid chromatography to a purity >98% to meet the requirements for in vitro and in vivo experiments. Specifically, peptides used in in vivo experiments featured amino acid sequences with D-isomer configurations. For in vitro studies, peptides were dissolved in PBS to prepare 10 mM stock solutions. For in vivo administration, K353-pe and K353R-pe were dissolved in PBS, stored on ice, and brought to room temperature immediately before injection.

To investigate the interventional effect of CPP on liver fibrosis, this study employed 3 classic models for mechanistic research: In CCl_4_- and MCD diet-induced liver fibrosis models, animals received daily intraperitoneal injections of K353-pe (50 mg/kg, dissolved in 200 μl of specific solvent) starting from the end of the second week until the end of the fourth week. For the BDL model, K353-pe intervention at the same dose and administration route was initiated on day 7 postsurgery and continued until day 14 postsurgery. Control groups were administered an equal volume of K353R-pe using identical dosage and injection protocols as the experimental groups. All animals were euthanized within 48 h after the last administration, and serum, liver, and other specimens were systematically collected for subsequent analysis.

### Statistical analysis

Statistically significant differences were assessed by 2-tailed unpaired Student *t* test or one-way analysis of variance (ANOVA) followed by Tukey’s Bonferroni or SNK multiple comparisons test. The results are presented as the mean ± SEM. The criterion for statistical significance was set at *P* < 0.05. Statistical analysis was performed using GraphPad PRISM 9.

## Data Availability

All data needed to evaluate the conclusions in the paper are present in the paper and/or the Supplementary Materials.
