# Supplementary material for: PGK1 Lactylation-Driven Self-Reinforcing Loop Orchestrates Glycolytic Reprogramming in FSP1+ Macrophages in Liver Fibrosis
Source: Research (Wash D C). 2026 Mar 3;9:1177. doi: 10.34133/research.1177 (PMC12953925; doi:10.34133/research.1177)
Supplement: Supplementary 1 — Figs. S1 to S10 Tables S1 and S2 [file research.1177.f1.docx]

Supplementary Materials for

**PGK1 lactylation-driven self-reinforcing loop orchestrates glycolytic reprogramming in FSP1^+^ macrophages in liver fibrosis**

Min Tang *et al.*

*Corresponding author: Changqing Yang, Email: cqyang@tongji.edu.cn, Fengshang Zhu, Email: zhufengshang@126.com, Hao Wang, Email: whdoublehai@126.com

**This PDF file includes:**

Figs. S1 to S10

Supplementary Materials

**
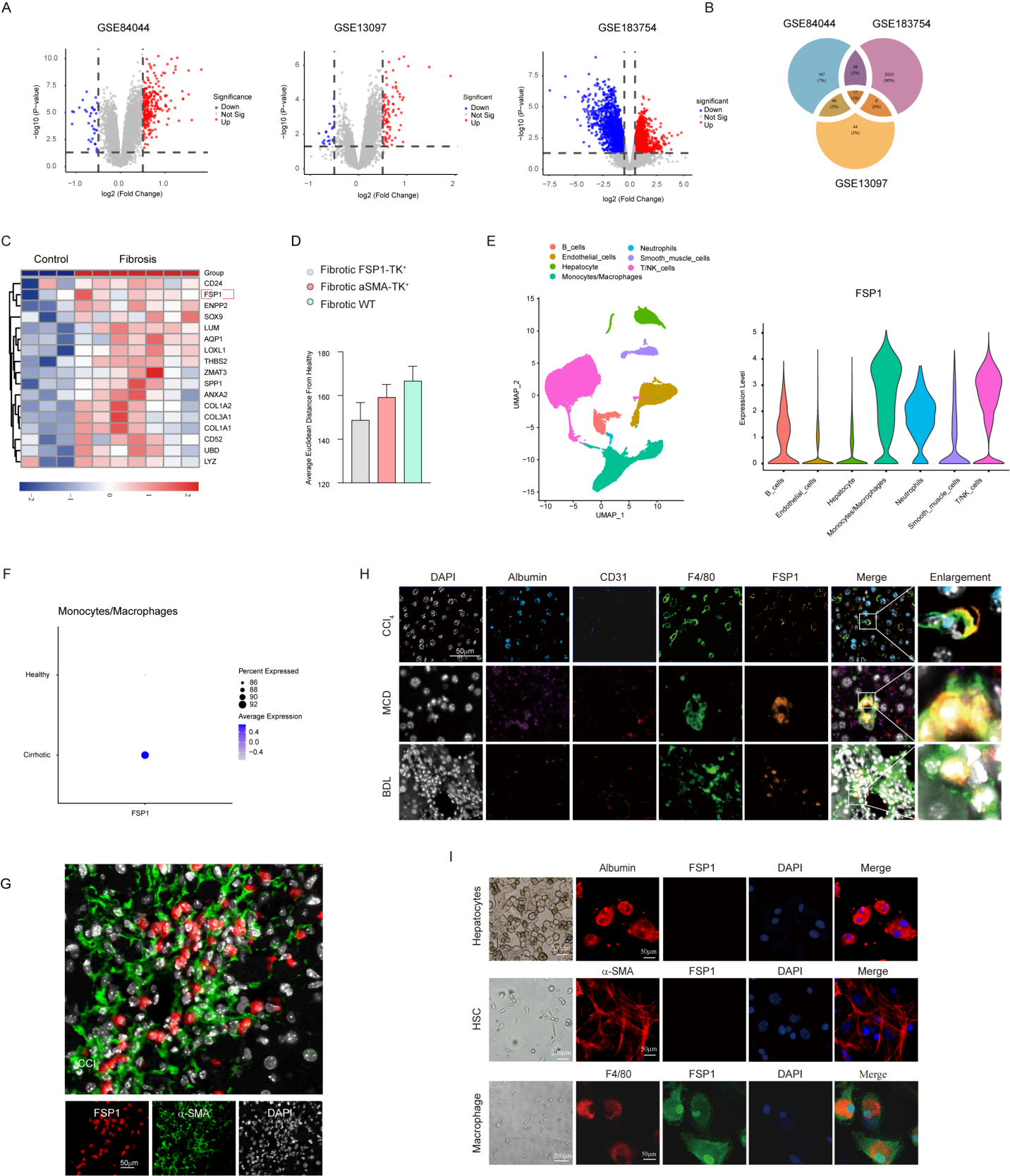
**

**Figure S1. FSP1 expression is increased in macrophage of liver fibrosis tissues.** (**A**) Volcano plots show gene expression changes between liver fibrosis and normal samples (pink: Upregulated, violet: downregulated; log₂FC > 0.5, P-values <0.05; gray: non-significant). (**B**) A Venn diagram illustrates overlapping upregulated genes among three datasets. (**C**) Heatmap displays expression patterns of 17 up-regulated genes common in GSE84044, GSE183754 and GSE130970. (**D**) The average Euclidean distance between the livers of aSMA-tk^+^ and FSP1-tk^+^ transgenic mice and healthy livers in the GSE55747 dataset. (**E**) UMAP plot of all liver cells (left) and expression of FSP1 (right) in liver (GSE136103). (**F**) Dot plot of FSP1 in monocyte/macrophage (GSE136103). (**G**) Dual-immunofluorescence staining of FSP1 and α-SMA in liver samples isolated from CCl4-induced liver fibrosis. Scale bar, 100μm. n = 6 per group. (**H**) Representative co-staining of FSP1 with hepatocytes (Albumin), endothelial cells (CD31^+^), macrophages (F4/80^+^) by mIHC in murine fibrotic liver tissue induced by the indicated treatments. n = 3 independent biological replicates. Scale bar, 50 μm. (**I**) Primary hepatocytes, HSCs and macrophages were isolated, and costaining of FSP1 and the marker of the primary cells. Scale bar, 50 μm in Immunofluorescence staining; 200μm in primary cells imaged under white light. n = 3 independent biological replicates.


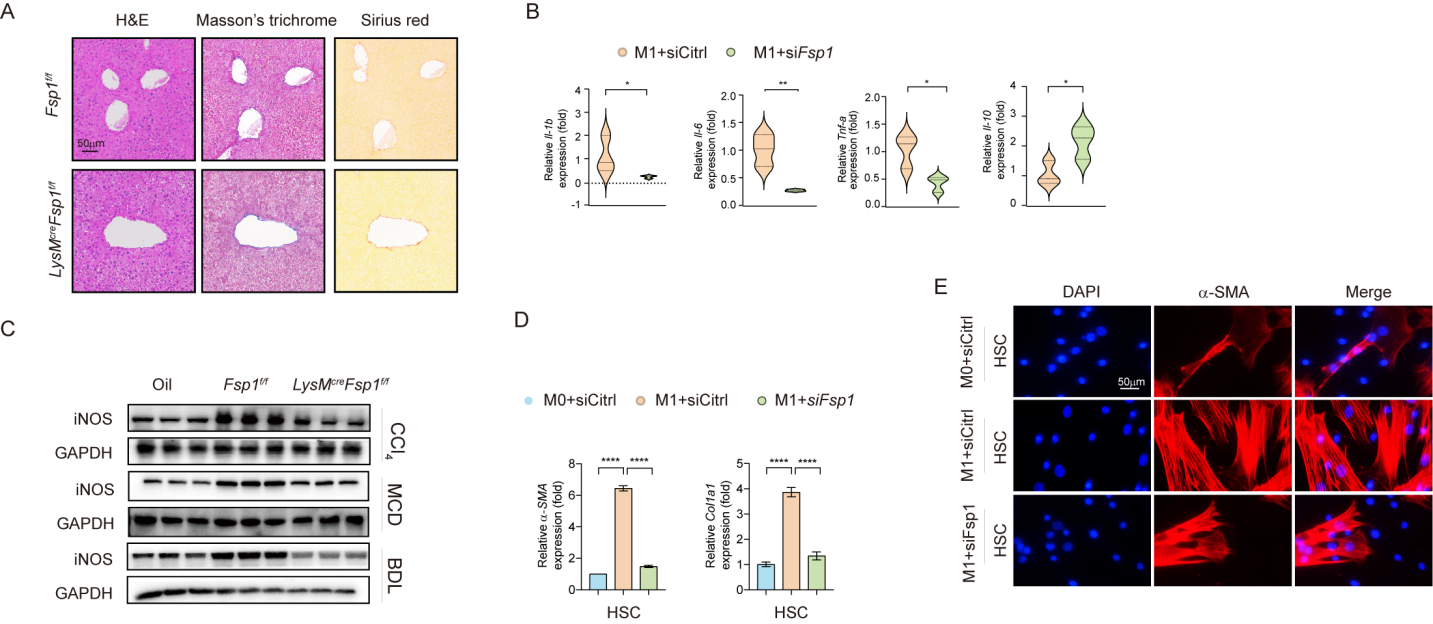


**Figure S2. FSP1^+^ cells are a subpopulation of macrophages during liver.** (**A**) Representative images of H&E, Sirius Red and Masson’s trichrome staining in liver sections of *Fsp1^f/f^* and *LysM^cre^Fsp1^f/f^* mice. scale bar, 50 μm. (**B**) mRNA expression of TNF-α, IL-1β, IL-6 and IL-10 was quantified in M1-polarized BMDM from the indicated group. (**C**) Western blot showed iNOS expression in liver tissues in the indicated groups. n = 6 per group. (**D**) BMDMs were treated with siCtrl or *siFsp1*. Primary HSCs were cocultured with BMDMs in an inflammatory environment induced by lipopolysaccharide (LPS)/interferon-γ (IFNγ), and α-SMA and col1α1 expression was analyzed by real-time PCR. n = 3 independent biological replicates. (**E**) α-SMA expression was analyzed by immunofluorescence staining in the indicated groups in (D). scale bar, 50 μm. Data were presented as mean ± SEM; Statistical significance was determined by unpaired Student’s t test (B) or one way ANOVA (D). **p* < 0.05, ***p* < 0.01.


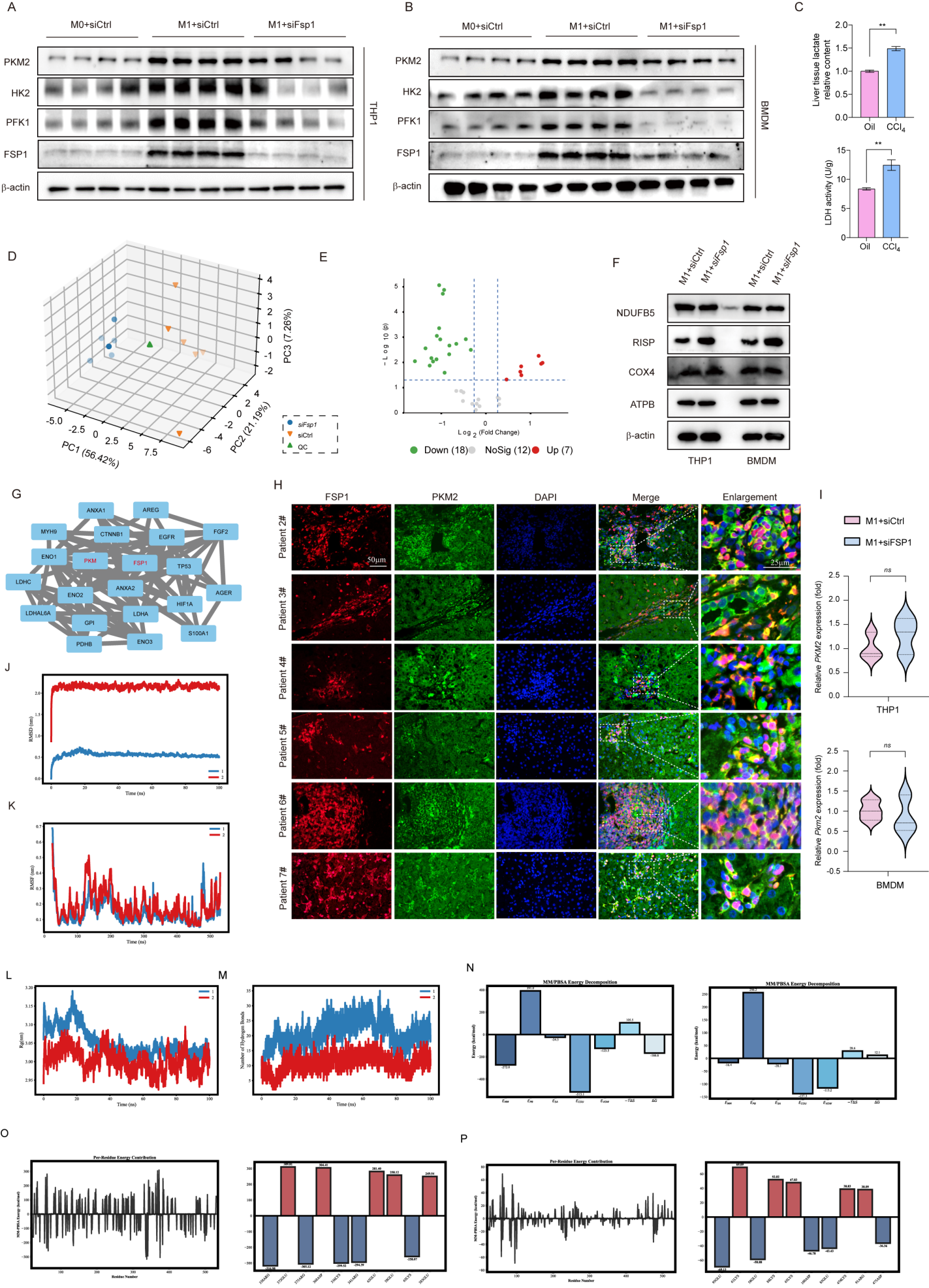


**Figure S3. FSP1 promotes glycolysis in macrophage by directly interacting with PKM2.** (**A-B**) Protein expression of PKM2, HK2 and PFK1 was analysed by Western blot analysis in BMDMs (A) and PMA-differentiated THP1 (B) tranfected with *siFsp1*. n = 4 per group. (**C**) Intracellular lactate and LDH activity were determined in CCl_4_ compared to healthy control mice. n = 6 per group. (**D**) PCA of metabolomics. n = 6 biological per group. (**E**) Volcano plots of the metabolomics with significant changes after FSP1 editing in M1-polarizated BMDMs. n = 6 per group. (**F**) Representative immunoblots of NDUFB5 (Complex I), RISP (Complex III), COX4 (Complex IV) and ATPB (Complex V) in BMDMs and PMA-differentiated THP1 receiving M1 or M1 with *siFsp1*. β-actin was selected as internal reference (bottom). n = 3 biological replicates per group. (**G**) Protein-Protein Interaction (PPI) of PKM2 and FSP1 was analyzed. (**H**) Representative immunofluorescence staining images of FSP1 (red) and PKM2 (green) in liver tissues from cirrhosis patient (n = 20). Scale bars, 50 μm. Enlargement, 25 μm. (**I**) The mRNA expression of PKM2 in M1-polarized BMDMs and THP1 after transfection with *siFsp1* or siCtrl. n = 3 biological replicates per group. (**J**) Root-mean-square deviation (RMSD) of the complex. (**K**) Root-mean-square fluctuation (RMSF) of the complex. (**L**) Radius of gyration (Rg) of the complex. **(M**) Number of hydrogen bonds (H-bonds) between the ligand and receptor proteins. (**N**) Binding free energy between the receptor and ligand proteins calculated by the MM/PBSA method. Left: System 1; Right: System 2. (**O-P**) Per-residue binding free energy decomposition for the complexes calculated by the MM/PBSA method. (O) System 1; (P) System 2. All data were mean ± SEM. Statistical significance was determined by unpaired Student’s t test. *ns*, not statistically significant; ***p* < 0.01 by Student’s t test.


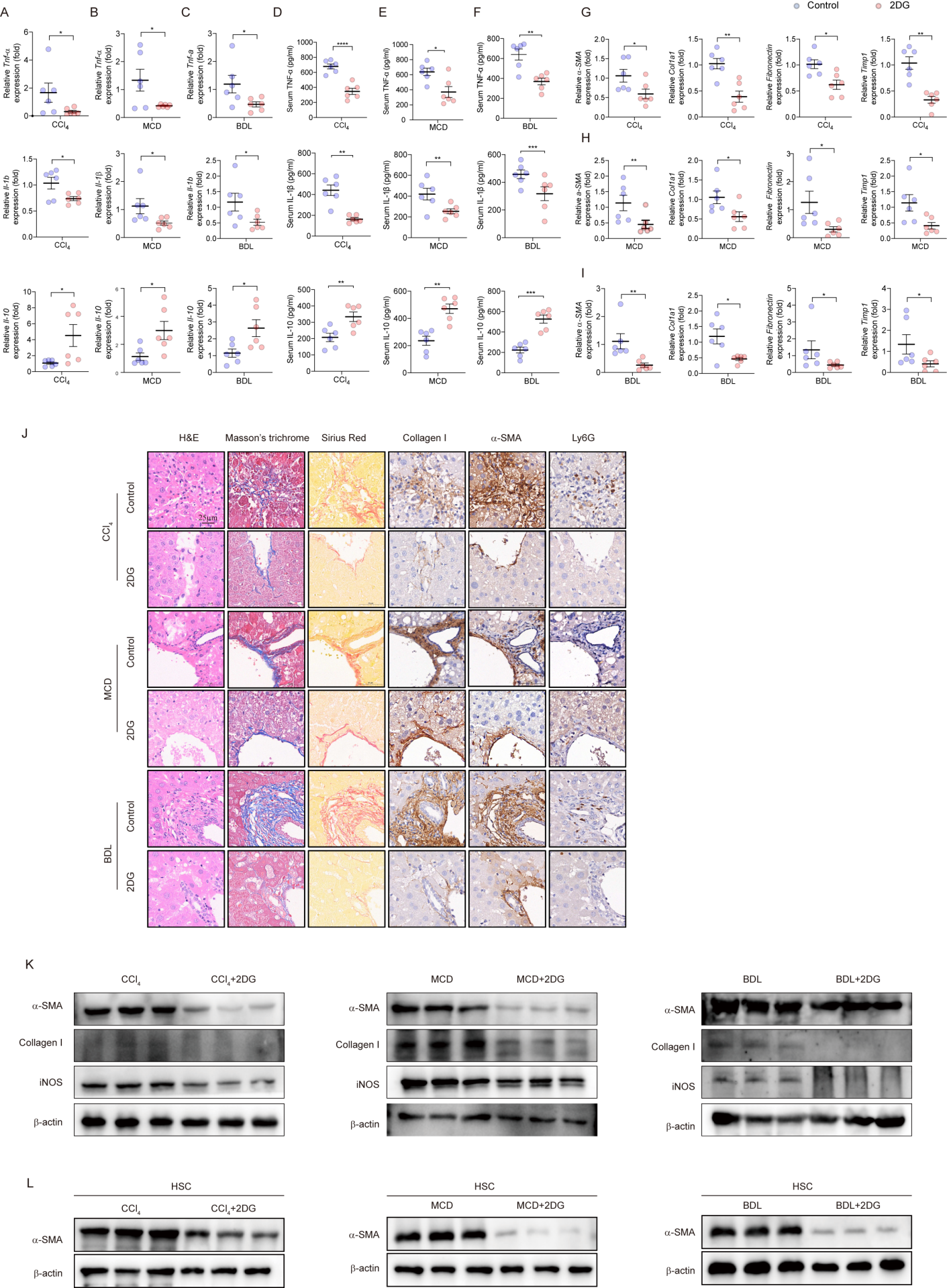


**Figure S4. Inhibition of glycolysis ameliorates liver fibrosis.** (**A-C**) The mRNA expression levels of *Tnf-α*, *Il-1β*, and *Il-10* were quantified in liver tissues from mice with fibrosis induced by CCl_4_, MCD diet, or BDL, followed by intraperitoneal injection of 2-DG. n = 6 per group. (**D-F**) Serum levels of TNF-α, IL-1β, and IL-10 were measured by ELISA in mice described in (A). (**G-I**) The mRNA expression of *α-SMA*, *Col1a1*, *Fibronectin* and *Timp* was quantified in liver tissues in the indicated groups. (**J**) The degree of fibrosis was evaluated by H&E, Masson’s trichrome, and Sirius Red staining, as well as immunohistochemical staining for collagen I and α-SMA. The degree of inflammation was evaluated by immunohistochemical staining for Ly6G in the indicated groups. scale bars, 50 μm. (**K**) Protein expression of collagen I, α-SMA, and iNOS was analyzed by Western blot in the indicated liver samples. n = 3 technical replicates per group. (**L**) Primary HSCs were isolated from the indicated groups, and α-SMA expression was measured by Western blot to evaluate HSCs activation. n = 3 technical replicates per group. Data were presented as mean ± SEM; Statistical significance was determined by unpaired Student’s t test. **p* < 0.05, ***p* < 0.01, ****p* < 0.001, *****p* < 0.0001. *ns*, no significant difference.


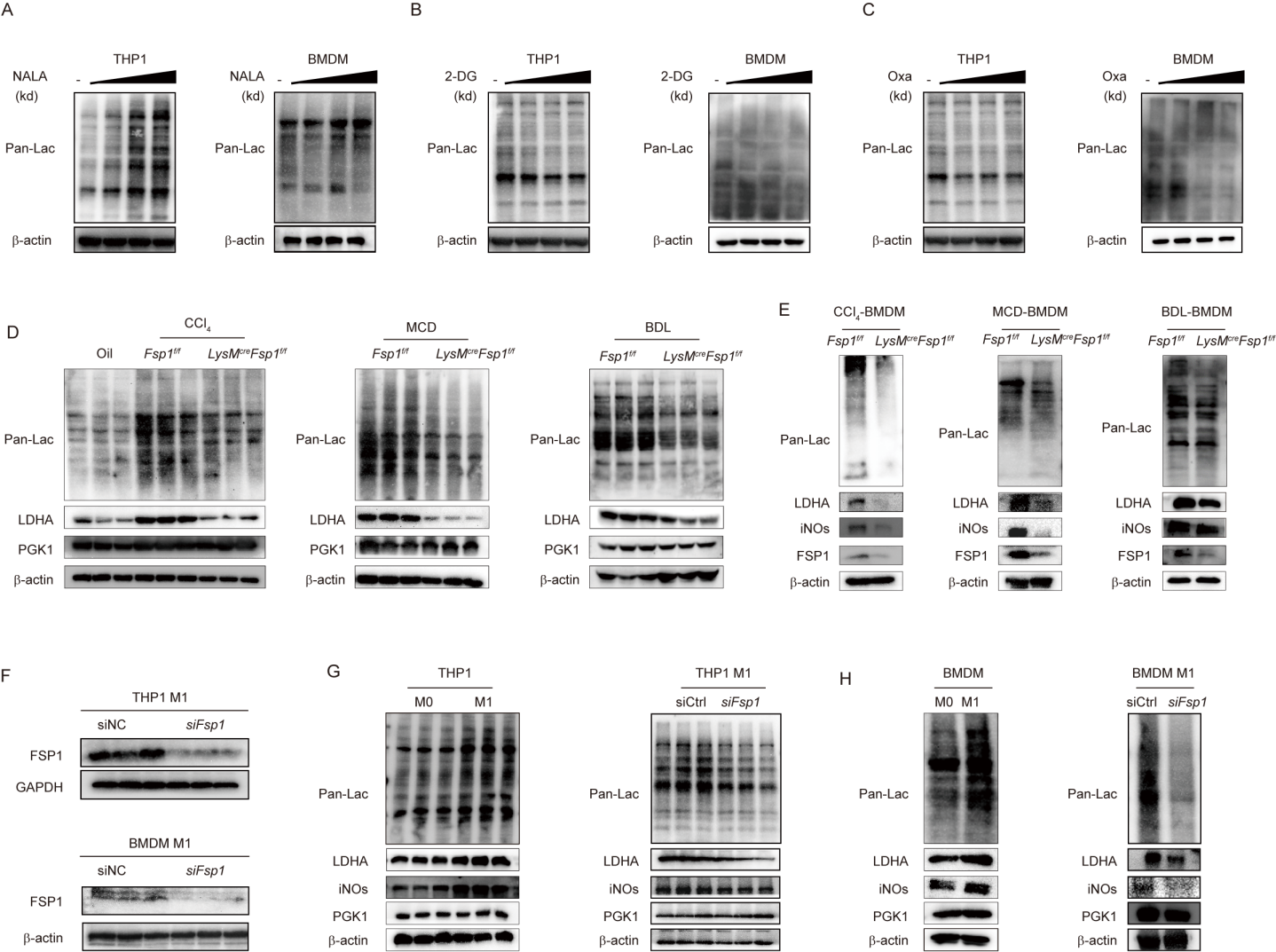


**Figure S5. FSP1-induced lactate accumulation drives protein lactylation modifications.** (**A**) PMA-differentiated THP1 and BMDMs were cultured with 10 mM, 25 mM, or 50 mM NALA for 24 hours. Total protein lactylation levels were evaluated by Western blot. n = 3 technical replicates per group. (**B**) PMA-differentiated THP1 cells and BMDMs were cultured in the presence of 10 mM, 25 mM, or 50 mM 2-DG for 24 hours. Total protein lactylation levels were evaluated by Western blotting analysis. n = 3 technical replicates per group. (**C**) PMA-differentiated THP1 cells and BMDMs were cultured in the presence of 5 mM, 10 mM, or 20 mM Oxamate (Oxa) for 24 hours. Total protein lactylation levels were evaluated by Western blotting analysis. n = 3 technical replicates per group. (**D**) Western blot analysis of total protein lactylation levels, LDHA, and PGK1 in liver tissues from control mice, *Fsp1^f/f^* and *LysM^cre^Fsp1^f/f^* mice with liver fibrosis. β-actin was used as a loading control (bottom panel). n = 3 technical replicates per group. (**E**) BMDMs were isolated from *Fsp1^f/f^* and *LysM^cre^Fsp1^f/f^* mice with CCl_4_-, MCD diet- and BDL-induced liver fibrosis. Total protein lactylation levels, the inflammatory factor iNOS, LDHA, and FSP1 were evaluated by Western blot. n = 3 technical replicates per group. (**F**) Western blot analysis was performed to measure FSP1 expression in PMA-differentiated THP1 and BMDMs after transfection with *siFsp1*. n = 3 technical replicates per group. (**G-H**) Total protein lactylation levels, the inflammatory factor iNOS, LDHA, and PGK1 were evaluated by Western blot in PMA-differentiated THP1 (G) and BMDMs (H) with the indicated treatment. n = 3 technical replicates per group.


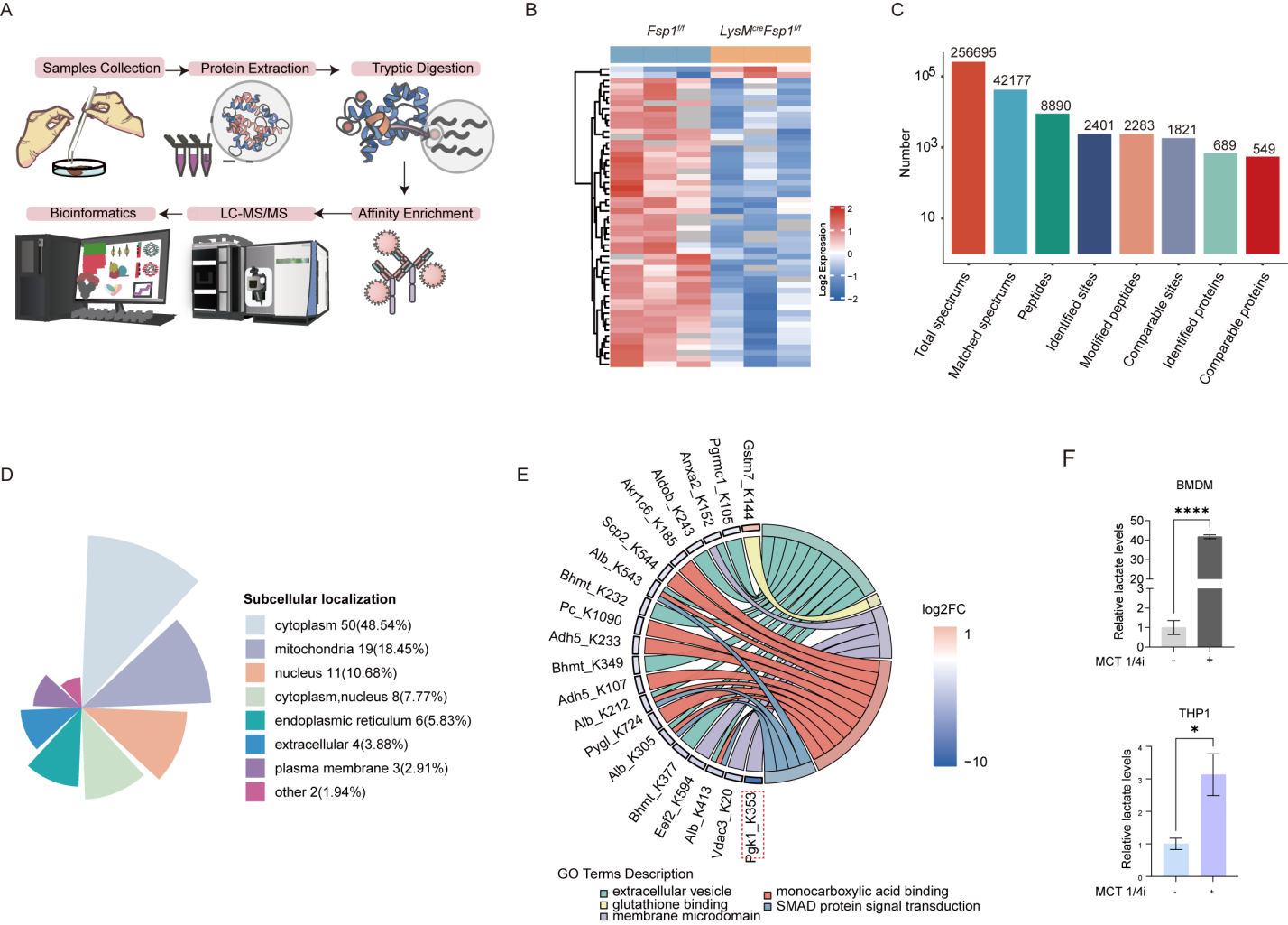


**Figure S6. Bioinformatic analyses of the differential proteome and lactylome.** (**A**) Liver tissues were collected from *Fsp1^f/f^* and *LysM^cre^Fsp1^f/f^* fibrotic mice induced by CCl_4_ injection (n=3), and the whole protein lysate samples were prepared. Samples were then subjected to immunoprecipitation using a pan lactic acidlysine antibody, followed by mass spectrometry for the precipitated constituents. (**B**) Heatmap displays proteins with statistically significant downregulation in lactylation levels from the samples identified in (A). (**C**) Data obtained in mass spectrometry (A) were further analyzed for numbers of lactylated sites, peptides and proteins. (**D**) Sunburst chart representing the subcellular distributions of Kla proteins. (**E**) The chord plot identified from differentially lactylated proteins between two groups and Gene Ontology (GO) terms of biological processes. (**F**) Measurement of intracellular lactate levels in THP1 and BMDMs pretreated with 20 mM MCT1/4i for 12 hours. Data were presented as mean ± SEM; Statistical significance was determined by unpaired Student’s t test. **p* < 0.05, *****p* < 0.0001.


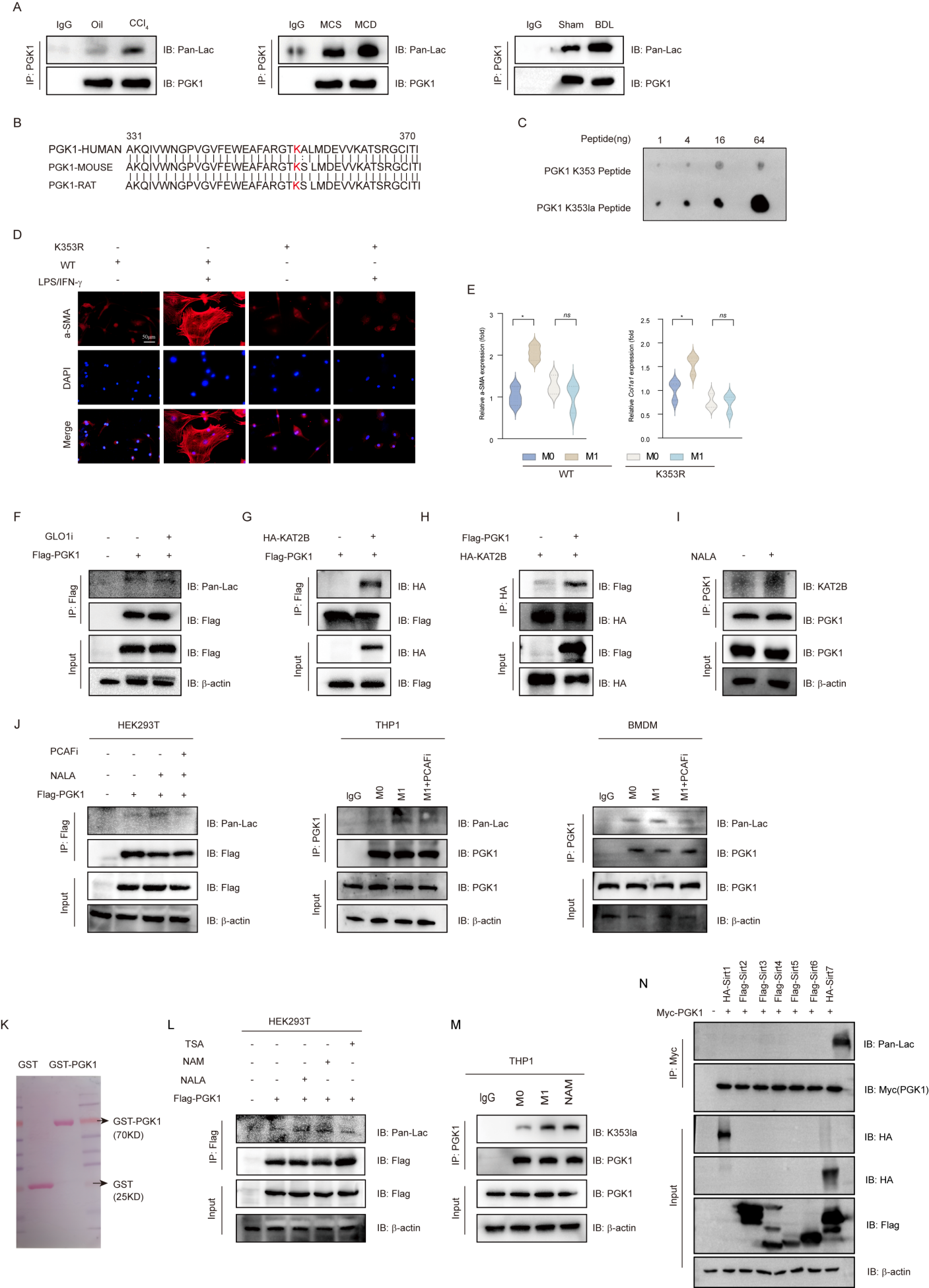


**Figure S7. PGK1 is lactylated at K353 by KAT2B lactyltransferase.** (**A**) The lactylation levels of PGK1 were measured in IP samples from fibrotic liver tissues induced by CCl_4_ injection, MCD diet, and BDL, compared to their respective controls. n = 3 technical replicates per group. (**B**) Protein sequence alignment of human, mouse and rat PGK1 using EMBOSS Matcher. (**C**) ddH2O containing different peptides was added onto the nitrocellulose membrane, followed by immunoblotting using anti-PGK1-K353la antibody. (**D-E**) M1 polarized BMDMs transfected with lentiviruses overexpressing WT PGK1 or the K353R mutant and induced to an M1 phenotype were co-cultured with primary HSCs. HSCs activation was assessed by fluorescence microscopy (D) and qRT-PCR analysis of *α-SMA* mRNA expression (E). (**F**) Lactylation of PGK1 was detected by IP in samples from HEK293T cells transfected with Flag-tagged PGK1 and treated with GLO1 inhibitor (GLO1i, 100μm) for 48 hours. n = 3 technical replicates per group. (**G-H**) CoIP assays were performed with anti-Flag M2 beads using HEK293T cells expressing Flag-tagged PGK1 and HA-tagged KAT2B, followed by immunoblotting with anti-HA and anti-Flag antibodies. n = 3 technical replicates per group. (**I**) The interaction of KAT2B and PGK1 was detected by CoIP in samples from BMDMs with NALA treatment. n = 3 technical replicates per group. (**J**) CoIP assays were performed with anti-Flag magnetic beads using HEK293T cells, PMA-differentiated THP-1 cells or BMDMs with PCAF inhibitor (PCAFi, 10 μM) for 24 hours. n = 3 biological replicates. (**K**) SDS-PAGE profile of purified GST fusion PGK1 WT. n = 3 biological replicates. (**L**) HEK293T cells expressing Flag-tagged PGK1 treated with NALA, NAM and TSA were subjected to CoIP assays using anti-Flag antibodies, followed by immunoblotting with anti-Pan-Lac and anti-Flag antibodies. (**M**) Lactylation of PGK1 was detected by CoIP in samples from PMA-differentiated THP-1 cells subjected to M1 polarization or NAM treatment for 24 hours. (N) Screening the “erasers” of PGK1 lactylation by transfecting combined Myc-tagged PGK1 and delactylase as indicated. Data were presented as mean ± SEM; Statistical significance was determined by unpaired Student’s t test. **p* < 0.05, *ns*, no significant difference.


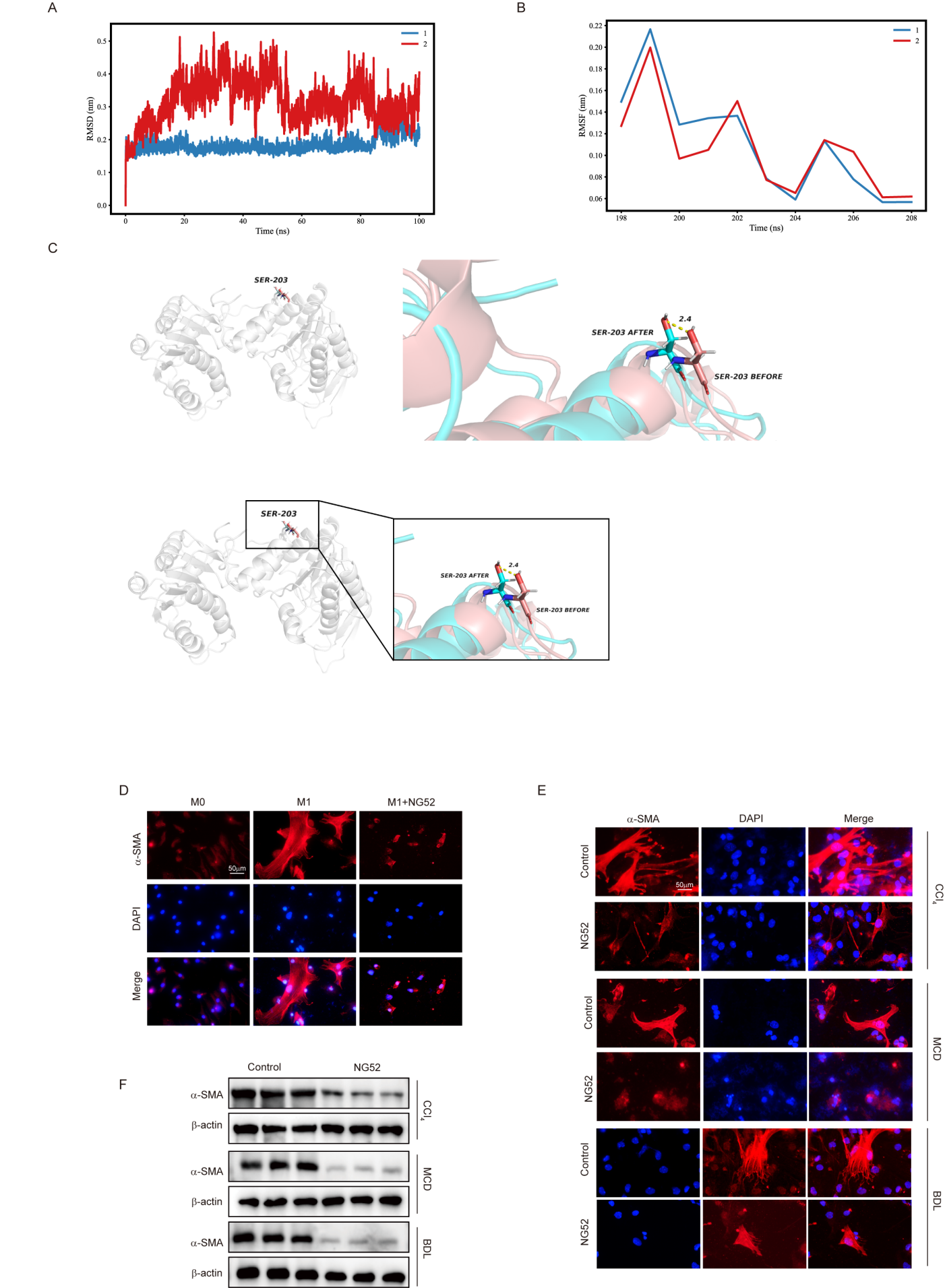


**Figure S8. NG52 treatment in macrophage inhibited HSCs activation. (A)** RMSD of the two systems. (**B)** RMSF of residues 198-208. (**C)** Snapshot of the final simulation frame. (**D**) BMDMs were induced to M1 polarization, followed by treatment with NG52 (a PGK1 inhibitor) or vehicle control. Co-cultured primary HSCs with BMDMs, α-SMA expression was analyzed by immunofluorescence staining. n = 3 biological replicates. Scale bars, 50 μm. (**E**) α-SMA expression was analyzed by immunofluorescence staining in primary HSCs isolated from fibrotic liver tissue with NG52 treatment or vehicle control. Nuclei were counterstained with DAPI. n = 6 per group. Scale bars, 50 μm. (**F**) α-SMA expression was analyzed by Western blot analysis in the samples described in (B).


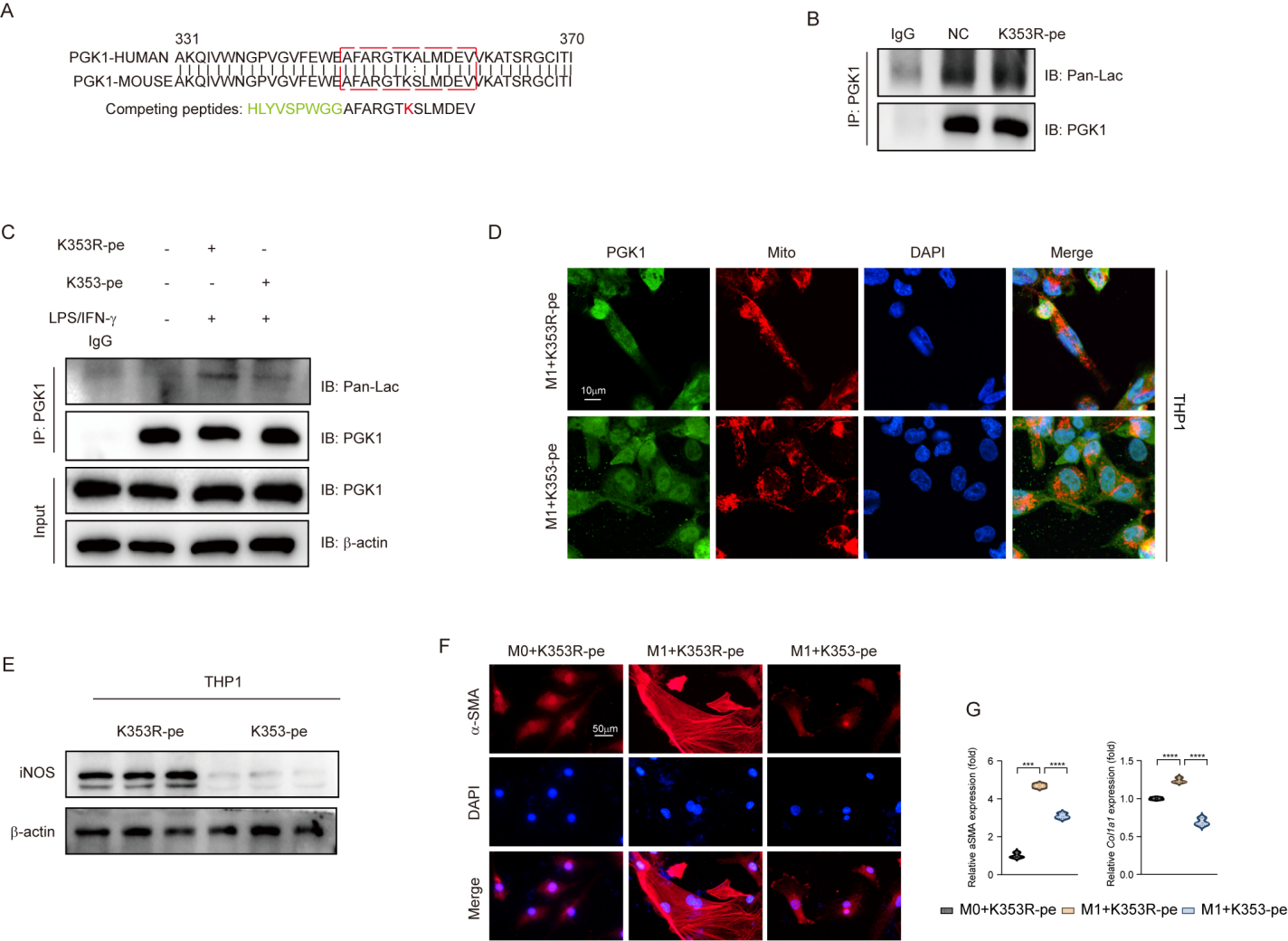


**Figure S9. Peptidic inhibitor decreased PGK1 lactylation. (A)** Schematic illustration of designed peptides. Green: cell-penetrating peptide (CPP). Red: site for PGK1 lactylation. (**B**) PGK1 lactylation was analyzed in BMDMs primed with LPS/IFNγ for 24 hours, followed by treatment with or without K353R-peptide (50 μM) for an additional 24 hours. IP analysis was performed to detect PGK1 lactylation and PGK1 expression. n = 3 biological replicates. (**C**)BMDMs were polarized to the pro-inflammatory M1 phenotype, and treated with K353R-peptide or K353-peptide for 24 hours. PGK1 lactylation was assessed by IP followed by Western blot analysis using a Pan-Lac and PGK1-specific antibody. n = 3 biological replicates. (**D**)M1-polarized THP1 were treated with K353-peptide or K353R-peptide, followed by staining with anti-PGK1 antibody and MitoTracker. Nuclei were counterstained with DAPI. n = 3 biological replicates. (**E**)Immunoblotting analysis of iNOS was assessed in THP1 cells treated with K353-peptide or K353R-peptide. n = 3 biological replicates. (**F-G**) BMDMs were induced to M1 polarization and treated with K353R-peptide or K353-peptide treatment. Co-cultured HSCs with BMDMs, α-SMA expression was analyzed by immunofluorescence staining (F) and qRT-PCR (G). n = 3 biological replicates. scale bars, 50 μm. All data were mean ± SEM. ****p* < 0.001, *****p* < 0.0001 by unpaired Student’s t test.


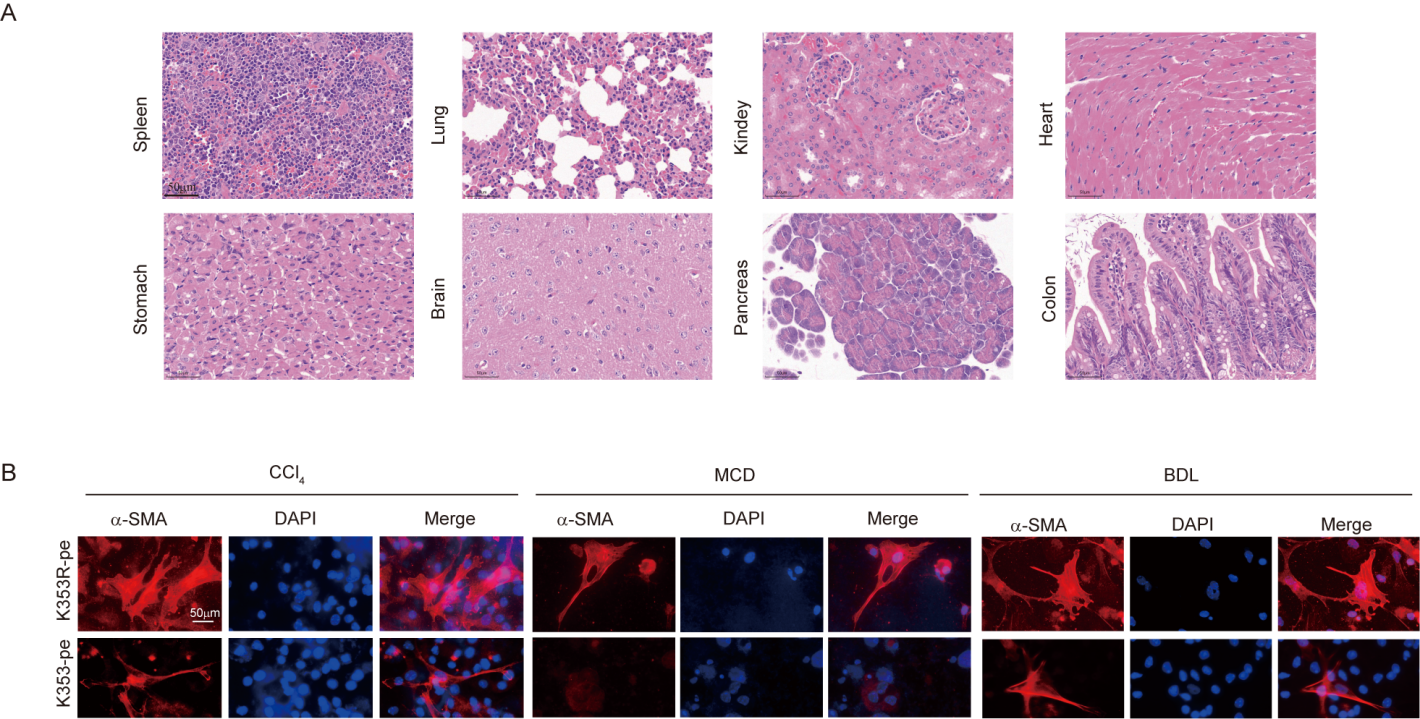


**Figure S10. Peptidic inhibitor alleviates the severity of liver fibrosis without obvious toxicity.** (**A**) The toxicity of CPP was evaluated by H&E staining in the indicated group. (**B**) α-SMA expression was analyzed by immunofluorescence staining in primary HSCs isolated from fibrotic liver tissue with the indicated treatment. Nuclei were counterstained with DAPI. n = 6 per group. Scale bars, 50 μm.

Supplementary Materials

Supplementary Table S1

| Table S1. Summary of Oligonucleotides used in the present study. | |  |
| --- | --- | --- |
| Oligonucleotides | Sequence (5'-3') | Source |
| mouse si-*Fsp1* | GGACAGATGAAGCTGCATT | Ribobio Biotech |
| human si-*Fsp1* | GCTTGATGCTGAGCAAGTT |  |
| si-KAT2B-1# | GCAGATACCAAACAAGTTTAT |  |
| si-KAT2B-2# | CGAACTCTAATCCTCACTCAT |  |
| mouse α-SMA Forward | GTCCCAGACATCAGGGAGTAA |  |
| mouse α-SMA Reverse | TCGGATACTTCAGCGTCAGGA | Sangon Biotech |
| mouse Col1α1 Forward | GCTCCTCTTAGGGGCCACT |  |
| mouse Col1α1 Reverse | CCACGTCTCACCATTGGGG |  |
| mouse TIMP1 Forward | CGAGACCACCTTATACCAGCG |  |
| mouse TIMP1 Reverse | ATGACTGGGGTGTAGGCGTA |  |
| mouse Fibronectin Forward | ATGTGGACCCCTCCTGATAGT |  |
| mouse Fibronectin Reverse | GCCCAGTGATTTCAGCAAAGG |  |
| mouse IL-1β Forward | GCAACTGTTCCTGAACTCAACT |  |
| mouse IL-1β Reverse | ATCTTTTGGGGTCCGTCAACT |  |
| mouse IL-10 Forward | GCTCTTACTGACTGGCATGAG |  |
| mouse IL-10 Reverse | CGCAGCTCTAGGAGCATGTG |  |
| mouse TNF-α Forward | CCCTCACACTCAGATCATCTTCT |  |
| mouse TNF-α Reverse | GCTACGACGTGGGCTCAG |  |
| mouse β-actin Forward | GGCTGTATTCCCCTCCATCG |  |
| mouse β-actin Reverse | CCAGTTGGTAACAATGCCATGT |  |
| human α-SMA Forward | TTCAATGTCCCAGCCATGTA |  |
| human α-SMA Reverse | GAAGGAATAGCCACGCTCAG |  |
| human Col1α1 Forward | ATCAACCGGAGGAATTTCCGT |  |
| human Col1α1 Reverse | CACCAGGACGACCAGGTTTTC |  |
| human TIMP1 Forward | CTTCTGCAATTCCGACCTCGT |  |
| human TIMP1 Reverse | ACGCTGGTATAAGGTGGTCTG |  |
| human Fibronectin Forward | AGGAAGCCGAGGTTTTAACTG |  |
| human Fibronectin Reverse | AGGACGCTCATAAGTGTCACC |  |
| human IL-1β Forward | GCTGGAGAGTGTAGATCCCAAA |  |
| human IL-1β Reverse | TGCTTGAGAGGTGCTGATGT |  |
| human IL-10 Forward | GACTTTAAGGGTTACCTGGGTTG |  |
| human IL-10 Reverse | TCACATGCGCCTTGATGTCTG |  |
| human TNF-α Forward | TGGCGTGGAGCTGAGAGATA |  |
| human TNF-α Reverse | TGATGGCAGAGAGGAGGTTG |  |
| human β-actin Forward | CATGTACGTTGCTATCCAGGC |  |
| human β-actin Reverse | CTCCTTAATGTCACGCACGAT |  |

| Table S2. Summary of antibodies used in Western blot/IHC/IF/flowcytometry. | | |
| --- | --- | --- |
| Recombinant Anti-FSP1 antibody [EPR14639(2)] | Abcam | Cat: ab197896 |
| FSP1 Antibody | Abmart | Cat: T55799 |
| Alpha smooth muscle actin Polyclonal antibody | Proteintech | Cat: 14395-1-AP |
| Alpha smooth muscle actin specific Monoclonal antibody | Proteintech | Cat: 67735-1-Ig |
| Collagen I alpha 1 Rabbit mAb | ZEN-bioscience | Cat: R26615 |
| Recombinant Anti-Collagen I antibody [EPR22894-89] | Abcam | Cat: ab260043 |
| GAPDH Polyclonal antibody | proteintech | Cat: 10494-1-AP |
| Recombinant Anti-CD11b antibody [EPR1344] | Abcam | Cat: ab133357 |
| iNOS Polyclonal antibody | Proteintech | Cat: 18985-1-AP |
| Albumin Polyclonal antibody | Proteintech | Cat: 16475-1-AP |
| Recombinant Anti-CD31 antibody [RM1006] | Abcam | Cat: ab281583 |
| F4/80 (D2S9R) XP® Rabbit mAb | Cell Signaling Technology | Cat: 70076 |
| PKM2-specific Polyclonal antibody | Proteintech | Cat: 15822-1-AP |
| PKM2-specific Monoclonal antibody | Proteintech | Cat: 60268-1-Ig |
| Hexokinase 2 Polyclonal antibody | Proteintech | Cat: 22029-1-AP |
| PFK1 Polyclonal antibody | Proteintech | Cat: 13389-1-AP |
| DYKDDDDK tag Polyclonal antibody (Binds to FLAG® tag epitope) | Proteintech | Cat: 20543-1-AP |
| Myc-Tag (19C2) mAb | Abmart | Cat: M20002S |
| HA-Tag(26D11) mAb | Abmart | Cat: M20003S |
| Recombinant ATPB Antibody | Aladdin | Cat: Ab090212 |
| NDUFB5 Antibody | Abmart | Cat: Ab156576 |
| Complex III Subunit 5 Rabbit mAb | Abmart | Cat: TN25597S |
| COX IV Antibody | Abmart | Cat: T55155 |
| Ly-6G (E6Z1T) Rabbit mAb | Cell Signaling Technology | Cat: 87048 |
| Anti-L-Lactyl Lysine Rabbit mAb | PTM Biolabs | Cat: PTM-1401RM |
| LDHA-Specific Polyclonal antibody | Proteintech | Cat: 19987-1-AP |
| PGK1 Polyclonal antibody | Proteintech | Cat: 17811-1-AP |
| PCAF Antibody (E-8) | Santa Cruz Biotechnology | Cat: sc-13124 |
| Anti-Acetyllysine Mouse mAb | PTM Biolabs | Cat: PTM-101 |
| Anti- Lactyl- PGK1 (K353) Rabbit pAb | PTM Biolabs | Cat: CO1149 |
| PDK1 Polyclonal antibody | Proteintech | Cat: 18262-1-AP |
| PDHK1(Phospho-Thr338) Antibody | Signalway Antibody | Cat: 11596 |
| PDH E1 Alpha Polyclonal antibody | Proteintech | Cat: 18068-1-AP |
| Phospho-PDH E1 Alpha (Ser293) Polyclonal antibody | Proteintech | Cat: 29580-1-AP |
| PGK1 (Phospho-Ser203) Antibody | Signalway Antibody | Cat: SAB487P |
| IPKine™ HRP, Mouse Anti-Rabbit IgG LCS | Abbkine | Cat: A25022P |
| beta Actin Recombinant Rabbit Monoclonal Antibody [PSH03-63] | Huabio | Cat: HA722023 |
| CoraLite488-conjugated Goat Anti-Mouse IgG(H+L) | Proteintech | Cat: SA00013-1 |
| CoraLite594 – conjugated Goat Anti-Rabbit IgG(H+L) | Proteintech | Cat: SA00013-4 |
| Multi-rAb™ CoraLite® Plus 488-Goat Anti-Rabbit Recombinant Secondary Antibody (H+L) | Proteintech | Cat: RGAR002 |
| HRP-labeled Goat Anti-Mouse IgG(H+L) | Beyotime | Cat: A0216 |
| HRP-conjugated Goat Anti-Rabbit IgG(H+L) | Proteintech | Cat: SA00001-2 |
| MHC Class II (I-A/I-E) antibody | Proteintech | Cat: FITC-65122 |
